# Secondary Metabolites, Biological Activities, and Industrial and Biotechnological Importance of *Aspergillus sydowii*

**DOI:** 10.3390/md21080441

**Published:** 2023-08-05

**Authors:** Sabrin R. M. Ibrahim, Shaimaa G. A. Mohamed, Baiaan H. Alsaadi, Maryam M. Althubyani, Zainab I. Awari, Hazem G. A. Hussein, Abrar A. Aljohani, Jumanah Faisal Albasri, Salha Atiah Faraj, Gamal A. Mohamed

**Affiliations:** 1 Preparatory Year Program, Department of Chemistry, Batterjee Medical College, Jeddah 21442, Saudi Arabia; 2 Department of Pharmacognosy, Faculty of Pharmacy, Assiut University, Assiut 71526, Egypt; 3 Faculty of Dentistry, British University, El Sherouk City, Cairo 11837, Egypt; shaimaag1973@gmail.com; 4 Department of Clinical Service, Pharmaceutical Care Services, King Salman Medical City, MOH, Al Madinah Al Munawwarah 11176, Saudi Arabia; bayanhs@hotmail.com (B.H.A.); ph.maryam91@gmail.com (M.M.A.); 5 Pharmaceutical Care Services, King Salman Medical City, MOH, Al Madinah Al Munawwarah 11176, Saudi Arabia; zawari.3000@outlook.com; 6 Preparatory Year Program, Batterjee Medical College, Jeddah 21442, Saudi Arabia; hazemgamal2005@gmail.com; 7 Pharmaceutical Care Services, Medina Cardiac Center, MOH, Al Madinah Al Munawwarah 11176, Saudi Arabia; ph1993abrar@gmail.com; 8 Pharmacy Department, Home Health Care, MOH, Al Madinah Al Munawwarah 11176, Saudi Arabia; jumanah.albasri@gmail.com; 9 Pharmacy Department, King Salman Medical City, MOH, Almadinah Almunawarah 11176, Saudi Arabia; salhafaraj66@gmail.com; 10 Department of Natural Products and Alternative Medicine, Faculty of Pharmacy, King Abdulaziz University, Jeddah 21589, Saudi Arabia; gahussein@kau.edu.sa

**Keywords:** fungi, *Aspergillus sydowii*, metabolites, enzymes, biotechnology, bioremediation, renewable resources, life on land, marine natural products, drug discovery

## Abstract

Marine-derived fungi are renowned as a source of astonishingly significant and synthetically appealing metabolites that are proven as new lead chemicals for chemical, pharmaceutical, and agricultural fields. *Aspergillus sydowii* is a saprotrophic, ubiquitous, and halophilic fungus that is commonly found in different marine ecosystems. This fungus can cause aspergillosis in sea fan corals leading to sea fan mortality with subsequent changes in coral community structure. Interestingly, *A. sydowi* is a prolific source of distinct and structurally varied metabolites such as alkaloids, xanthones, terpenes, anthraquinones, sterols, diphenyl ethers, pyrones, cyclopentenones, and polyketides with a range of bioactivities. *A. sydowii* has capacity to produce various enzymes with marked industrial and biotechnological potential, including α-amylases, lipases, xylanases, cellulases, keratinases, and tannases. Also, this fungus has the capacity for bioremediation as well as the biocatalysis of various chemical reactions. The current work aimed at focusing on the bright side of this fungus. In this review, published studies on isolated metabolites from *A. sydowii*, including their structures, biological functions, and biosynthesis, as well as the biotechnological and industrial significance of this fungus, were highlighted. More than 245 compounds were described in the current review with 134 references published within the period from 1975 to June 2023.

## 1. Introduction

Fungi have so far received substantial attention for enhancing value in agricultural, industrial, pharmaceutical, and health fields [1,2,3,4]. During the past few decades, there have been some extremely intriguing advances in the utilization of fungi for new processes, products, and solutions that are crucial for the world. Also, fungi are proven to be a prolific pool of structurally varied bioactive metabolites. Additionally, fungal enzymes have been utilized instead of chemical processes in various industries, including those of textiles, leather, paper, pulp, animal feed, baked goods, beer, wine, and juice, which greatly reduces negative environmental effects [5]. Genus *Aspergillus* (Moniliaceae) is one of the most valuable fungal genera of commercial, biotechnological, and medicinal importance [6,7,8]. It comprises 400 species and attracts remarkable interest as a wealthy pool of structurally varied metabolites, including terpenoids, alkaloids, peptides, xanthones, and polyketides [7,8,9]. These metabolites have diverse bioactivities such as antibacterial, cytotoxic, antifungal, and anti-HIV activities.

*Aspergillus sydowii* is a saprotrophic, ubiquitous, and halophilic fungus and represents one of the widely distributed *Aspergillus* species [10,11,12]. It is commonly found in different habitats all over the world, including diverse soil and marine ecosystems, and possesses a broad range of salinity tolerance [13]. Interestingly, halophilic *A. sydowii* is employed as a model organism for investigating filamentous fungi’s molecular adaptation to hyperosmolarity [13]. *A. sydowii* can survive as a food contaminant, as a soil-decomposing saprotroph, and as an opportunistic human pathogen [14]. It causes onychomycosis and aspergillosis in humans, as well as aspergillosis in sea fan corals, on the basis of Koch’s postulate and physiological, morphological, and nucleotide sequence analyses [15,16,17]. Aspergillosis symptoms involve small necrotic lesions of tissues with purple halos, like the pathology of coral bleaching [18]. This leads to sea fan mortality and subsequent changes in coral community structure [18]. It was reported to cause 20–90% mortality in sea fans in the Florida Keys [18].

In addition to its pathogenic potential, *A. sydowii* has captured a considerable number of researchers’ attention due to its capacity to create a variety of biotechnologically and industrially significant enzymes, such as lipases, α-amylases, xylanases, cellulases, tannases, and keratinases [19,20,21,22,23]. Additionally, *A. sydowii* biosynthesizes various classes of metabolites, such as sesquiterpenoids, alkaloids, xanthones, monoterpenes, anthraquinones, sterols, triterpenes, diphenyl ethers, pyrones, cyclopentenones, anthocyanins, and polyketides [11,24,25,26,27,28,29,30,31,32,33,34,35,36,37,38]. These metabolites have drawn remarkable interest because of their prominent bioactivities, including antioxidant, immunosuppression, antiviral, anti-mycobacterial, antimicrobial, cytotoxic, anti-inflammation, protein tyrosine phosphatase 1B (PTP1B) inhibition, anti-nematode, anti-diabetic, and anti-obesity properties [32,34,37,39,40,41,42,43,44,45,46,47,48]. Further, this fungus is employed for the synthesis of different types of nanoparticles that could have beneficial pharmaceutical, biotechnological, and industrial applications [49,50,51,52]. Recently, the number of articles on *A. sydowii* metabolites and their biotechnological and industrial relevance has risen substantially. It is noteworthy that a review paper discussing *A. sydowii*, particularly the positive aspects of this commercially useful fungus, was not found. Therefore, the current work provided a comprehensive and close insight into this fungus. The published information on the secondary metabolites identified from this fungus and their bioactivities were compiled. Additionally, the research on *A. sydowii*, including applications in industry, biotechnology, and nanotechnology, has been reviewed. Studies published in the literature within the period from 1975 to 2023 were reported. Additionally, the documented biosynthesis routes of the fungus’ major metabolites were illustrated.

Searches were conducted in depth on literature databases, namely PubMed, Web of Science, and Scopus, as well as on various websites of publishers (Wiley Online Library, Taylor & Francis, Springer, JACS, Thieme, and Bentham) and scientific websites (Google Scholar, PubMed, and ScienceDirect). The following phrases and keywords were used for the search: “*Aspergillus sydowii*,” “*Aspergillus sydowii* + compounds,” “*Aspergillus sydowii* + metabolites,” “*Aspergillus sydowii* + NMR,” “*Aspergillus sydowii* + biological activity,” “*Aspergillus sydowii* + Enzymes,” “*Aspergillus sydowii* + biotechnology,” “*Aspergillus sydowii* + biotechnological importance,” and “*Aspergillus sydowii* + nanoparticles”.

## 2. Secondary Metabolites of *Aspergillus sydowii*

### 2.1. Sesquiterpenes

Phenolic bisabolane sesquiterpenoids are among the main constituents reported from this fungus. They are a rare class of terpenes that have a p-alkylated benzene connected with 1C and 8C side chains at C-5 and C-2, respectively. Their structural variability is due to cyclization, reduction, or oxidation at various alkyl chain carbons to yield carboxylic acid, alcohol, lactone, double bond, pyran, and furan functionalities. Besides their fascinating skeletons, they show various bioactivities. It is noteworthy that most of the reported bisabolanes were separated from marine-derived *A. sydowii* as discussed below (Table 1). 

**Figure 1:**
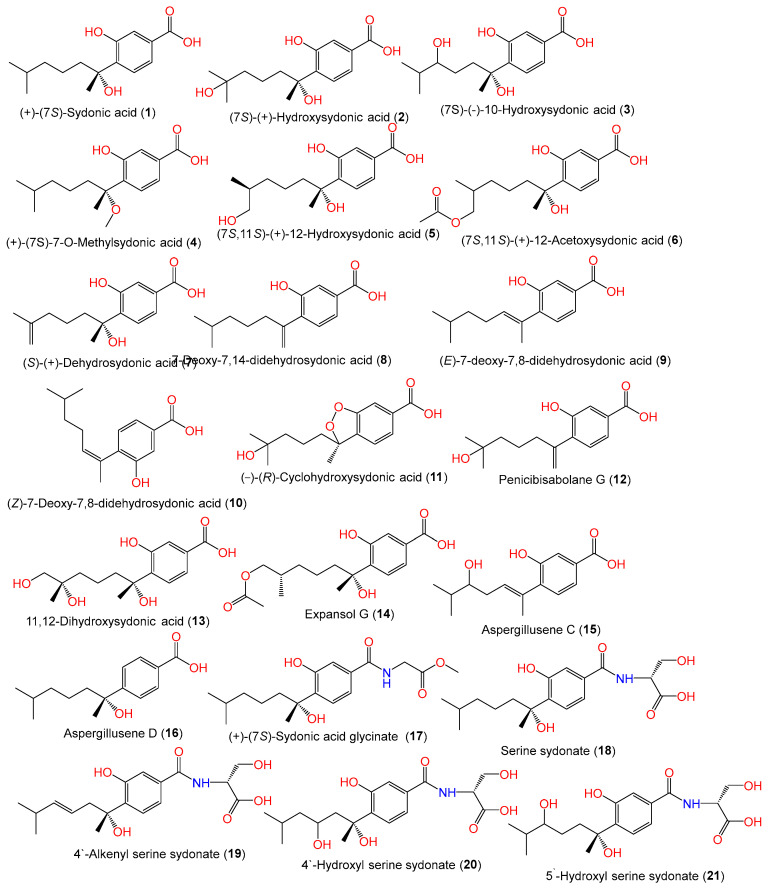
Structures of sesquiterpenoids (**1**–**21**) reported from *A. sydowii.*

In 1978, Hamasaki and his group separated and characterized compounds **1** and **2** as optically inactive metabolites from *A. sydowii* acetone extract by spectral and chemical means. These compounds were soluble in saturated NaHCO_3_ and positively reacted with bromophenol blue [11] (Figure 1).

Aspergillusene D (**16**) with a 7*S*-configuration was reported as a new sesquiterpenoid from *Phakellia fusca*-associated *A. sydowii* SCSIO-41301 by Liu et al., along with compounds **1**, **5 8**, **9**, **10**, and **22** that were characterized based on spectral and ECD (electronic circular dichroism) analyses [35]. Xu et al. separated compound **17**, along with compounds **1**, **8**, and **23**, from *A. sydowii* CUGB-F126 isolated from the Bohai Sea, Tianjin, using SiO_2_ (silica gel)/Sephadex LH-20/HPLC (high-performance liquid chromatography). Compound **17** is a new sydonic acid analog with a glycinate moiety [15]. 

**Figure 2:**
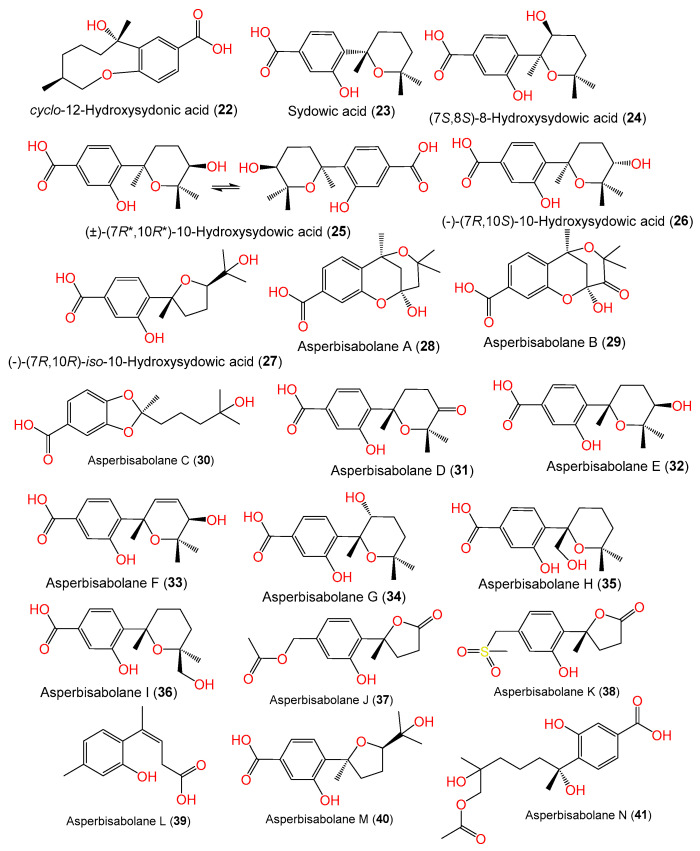
Structures of sesquiterpenoids (**22**–**41**) reported from *A. sydowii*.

Sun et al. developed a new approach that integrated computational programs (MS (mass spectrometry)-DIAL and MS-FINDER) and web-based tools (MetaboAnalyst and GNPS) for the identification of *A. sydowii*–*Bacillus subtilis* coculture metabolites, wherein 25 biosynthesized metabolites were detected and purified by SiO_2_/ODS CC/HPLC. Among them, compounds **1**, **2**, **3**, and **18**–**21** were characterized by spectral and CD (circular dichroism) analyses [58]. Further, Hu et al. separated and characterized new bisabolene-type sesquiterpenoids **24** and **25** as well as the known analogs **2** and **23** from *A. sydowii* EN-434 obtained from *Symphyocladia latiuscula* marine red alga using RP-18 (reversed phase-18)/SiO_2_ CC (silica gel column chromatography) and spectral and ECD data. Compounds **24** and **25** have 7*S*/8*S* and 7R*/10R* configurations, respectively [32]. Fourteen new phenolic bisabolanes with varied structures, labeled **28**–**41**, were separated and characterized by Niu et al. from the deep-sea sediment-derived *A. sydowii* MCCC-3A00324 (Figure 2). 

Compounds **28** and 29 are the first bisabolanes with a 6/6/6 tricyclic skeleton, whereas compound **30** features a novel *seco*-bisabolane with a rare dioxolane moiety, and compound **38** has an unusual methylsulfonyl moiety [57]. Trisuwan et al. purified—from *A. sydowii* PSUF154 isolated from gorgonian sea fan of genus *Annella*—new bisabolane-type sesquiterpenes **4**, **42**, and **43**, along with **1**. Compound **42** has 2-substituted 6-methyl-2-heptenyl and 1,2,4-trisubstituted benzene. Compound **43**‘s benzofuran moiety results from the ether linkage of C-1 OH of the tri-substituted phenyl and 2-substituted 6-methyl-2-heptenyl moieties. Compound **4** is a methyl ether of compound **1** with a 7*S* configuration [56]. The first phenolic bisabolane sesquiterpene glycoside, *β*-D-glucopyranosyl aspergillusene A (**44**), was purified from sponge-derived *A. sydowii* [36] and assigned using spectral and chemical methods [36].

**Scheme 1:**
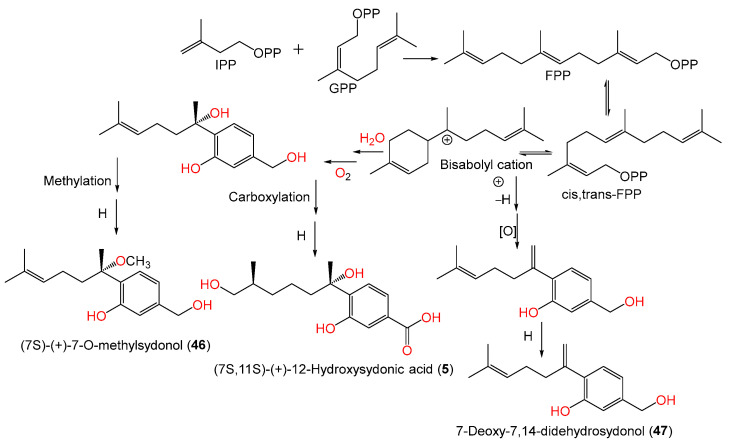
Biosynthetic pathway of compounds **5**, **46**, and **47**: GPP: Geranyl diphosphate; FPP: Farnesyl diphosphate; IPP: Isopentenyl diphosphate [54].

**Figure 3:**
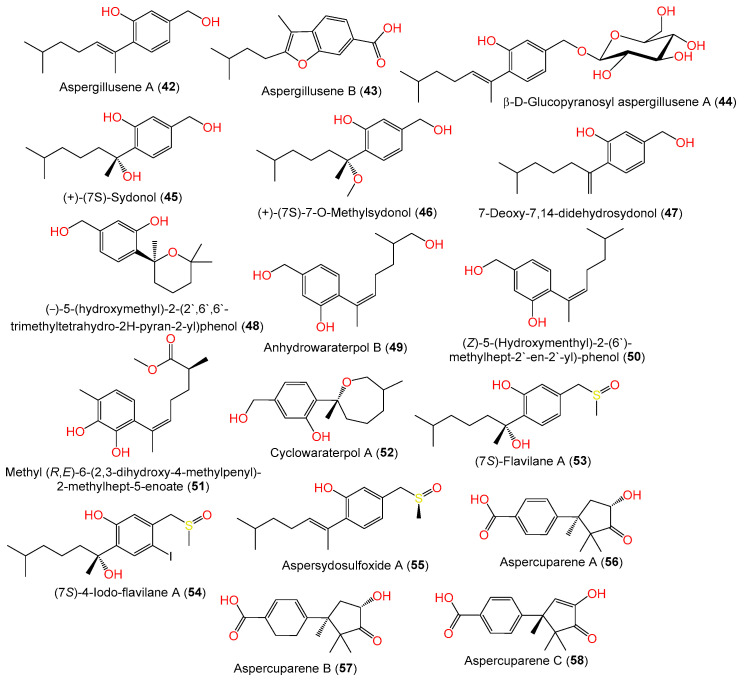
Structures of sesquiterpenoids (**42**–**58**) reported from *A. sydowii*.

Chung et al. stated that the addition of 5-azacytidine (a DNA methyltransferase inhibitor) to the culture of marine sediment-derived *A. sydowii* obtained from Hsinchu, Taiwan, significantly promoted the production of various metabolites [54]. Investigation of the EtOAc (ethyl acetate) extract of 5-azacytidine-treated culture broth by SiO_2_ CC and HPLC yielded new bisabolane sesquiterpenoids **5**, **46**, and **47**, along with **1**, **42**, **45**, and **49**, that were assigned based on spectral analyses. The S-configuration of compounds **5** and **46** was assigned using optical rotation comparison, whereas compound **46** ([α]D +1.87) is a methyl derivative of compound **45** ([α]D +7.2) and compound **5** ([α]D +3.9) is C-12 hydroxy analog of compound **1** ([α]D +23) (Figure 3). On the other hand, compound **47** is closely similar to the previously reported compound **8** except for the absence of the C-3 carboxylic group in compound **47** [54]. Compounds **5**, **46**, and **47** were proposed to be biosynthesized from farnesyl diphosphate (FPP) created from the addition of an IPP (isopentenyl diphosphate) unit to a GPP (geranyl diphosphate) (Scheme 1). Then, cyclization and folding of the carbon chain through an electrophilic attack on double bonds produced the bisabolane nucleus that then underwent a series of carboxylation, hydration, oxidation, and reduction to give compounds **5**, **46**, and **47** [54].

A new bisabolane sesquiterpenoid, compound **15**, in addition to compounds **1**, **7**, **6**, **42**, **47**, **49**, **50**, and **52**, were purified from *A. sydowii* ZSDS1-F6 EtOAc extract using SiO_2_/Sephadex LH-20/RP-HPLC by Wang et al. [45]. Compound **51** is a new aromatic bisabolene-type sesquiterpenoid with 11S-configuration purified and characterized from the sea-derived *A. sydowii* SW9 [41]. In 2022, Liu et al. purified a rare iodine- and sulfur-containing derivative (7*S*)- 4-iodo-flavilane A (**54**) along with compound **53**. Compound **54** is 4-iodinated analog of compound **53** and its absolute S-configuration was proven by ECD analysis [38]. Furthermore, three undescribed cuparene-type sesquiterpenes, labeled **56**–**58**, were isolated from fermented cultured EtOAc extract of the sea sediment-derived *A. sydowii* MCCC-3A00324 using SiO_2_/RP-18/Sephadex LH-20 CC/HPLC and assigned using spectral and ECD analyses. They represent rare cuparene-type sesquiterpenoids having a C-10 keto group and were discovered for the first time from filamentous fungi [57]. 

### 2.2. Mono- and Triterpenoids and Sterols

**Figure 4:**
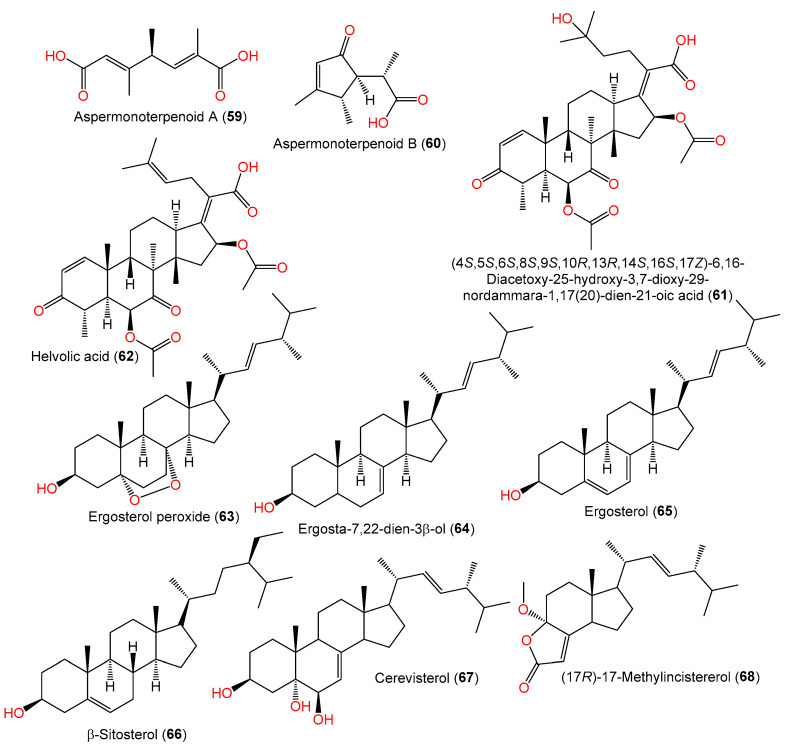
Structures of mono- (**59** and **60**) and triterpenoids (**61** and **62**) and sterols (**63**–**68**) reported from *A. sydowii.*

In 2020, the chemical investigation of deep-sea sediment-isolated *A. sydowii* MCCC-3A00324 by Niu et al. led to the separation of new osmane-type monoterpenoids aspermonoterpenoids A (**59**) and B (**60**) by SiO_2_ CC/HPLC and their structures were determined by spectral, ECD, and specific rotation analyses (Table 2, Figure 4). Compounds **59** and **60** are the first osmane monoterpenes reported from fungi, whereas compound **59** features a novel skeleton, which is possibly derived after the cleavage of the cyclopentane ring and oxidation reaction of the osmane monoterpenoid. They have 4*S* and 4*S*/5*R*/6*S* configurations, respectively [60]. 

**Scheme 2:**
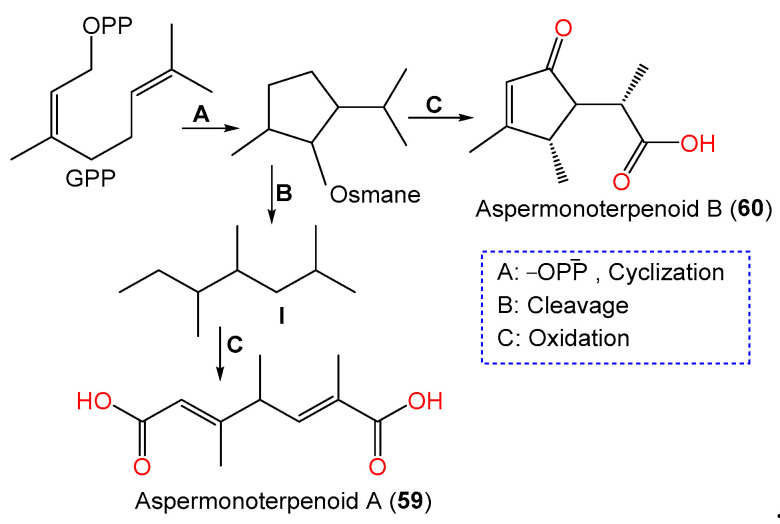
Biosynthetic pathway of compounds **59** and **60** [60].

These metabolites were proposed to be biosynthesized from a GPP that underwent subsequent hydrolysis/oxygenation/cyclization to yield the monocyclic osmane monoterpenoid ring. Then, carbon–carbon bond cleavage of osmane gives intermediate **I** and its further oxygenation yields compound **59**, whilst the osmane oxygenation forms compound **60** [60] (Scheme 2).

Zhang et al. purified and characterized compound **61**, a new 29-nordammarane-type triterpenoid, in addition to its known analog, compound **62**, from the marine-derived *A. sydowii* PFW1-13 [48]. Compound **61** is structurally similar to compound **62** with a 1,1,2-trisubstituted ethanol unit instead of a trisubstituted ethenyl unit, suggesting that compound **61** is a C_24_–C_25_ hydrated derivative of compound **62** [48]. Its configuration was assigned as 4*S*/5*S*/6*S*/8*S*/9*S*/10*R*/13*R*/14*S*/16*S*/17*Z* based on comparing its optical rotation (−118.9) with that of compound **62** (optical rotation −105.1) [48].

Wang et al., in 2019, reported the separation of ergosterol derivatives **63**–**66** from deep-sea water-isolated *A. sydowii* [55], while compounds **68** and **69** were separated by Li et al.; compound **69** was assumed to be a sterol degradation product [44].

### 2.3. Xanthone and Anthraquinone Derivatives

Xanthones are commonly found in lichen, fungi, plants, and bacteria [61]. In fungi, xanthones are mostly derived from acetyl-CoA through a series of polyketide synthase-catalyzed chemical transformations [62]. These metabolites were found to demonstrate diverse bioactivities.

**Figure 5:**
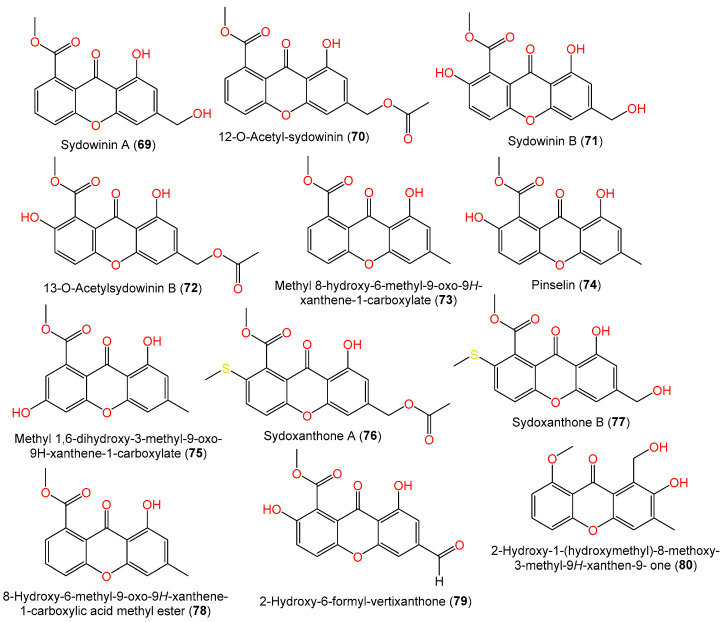
Structures of xanthones (**69**–**80**) reported from *A. sydowii.*

Compounds **69** and **71** were reported from the EtOAc extract of 5-azacytidine-treated *A. sydowii* culture broth [54]. Additionally, from liverwort *Scapania ciliate*-accompanied *A. sydowii*, new xanthone derivatives, labeled **72**, **76**, and **77**, and known compounds **74** and **78** were isolated by SiO_2_/Sephadex LH-20 CC/HPLC and assigned by spectral data. Compounds **76** and **77** are examples of sulfur-containing xanthones; compound **77** has an additional acetyl group at C-13 and compound **72** features C-2-OH instead of the methylthio moiety as in compound **76** [63]. New hydrogenated xanthones, aspergillusones A (**86**) and B (**87**), along with compounds **69**, **71**, **73**, **88**, and **90**, were purified from a strain associated with the gorgonian sea fan of the genus *Annella* by Trisuwan et al. Compound **86** is a 11-deoxy derivative of compound **88** with an optical rotation of −1.6 and the same C-7 and C-8 absolute configuration, whereas compound **87** is a 1-hydroxy analog of compound **90** with 7R/8R and −46.3 optical rotation (Figure 5) [56].

In 2019, Wang et al. purified two novel xanthones, labeled **70** and **79**, along with the known xanthones **71**, **72**, **74**, **86**, **88**, and **89** and quinones **91**, **94**, and **96**, from seawater-derived *A. sydowii* C1-S01-A7 using SiO_2_/Sephadex LH-20/RP-18/HPLC; the compounds were elucidated by spectral analyses (Table 3). Compound **79** is similar to previously reported 2-hydroxyvertixanthone with an additional formyl moiety at C-6, whereas compound **70** is similar to compound **69** with one more acetyl group at C-12 [55]. 

**Figure 6:**
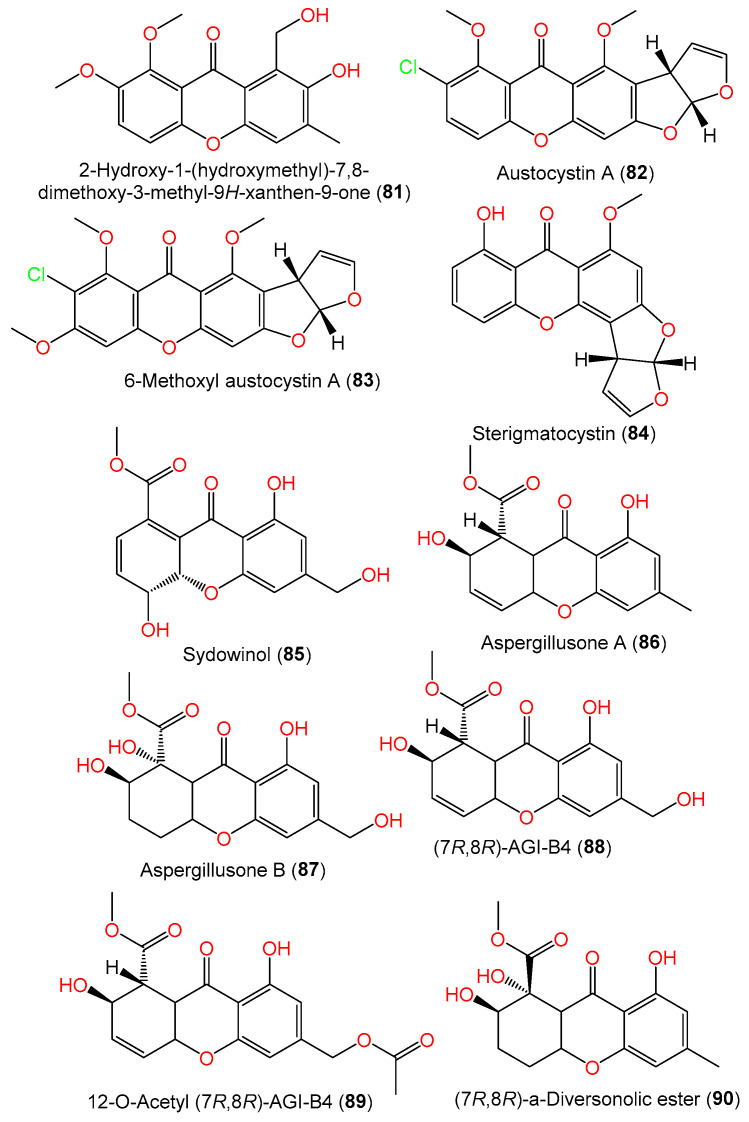
Structures of xanthones (**81**–**90**) reported from *A. sydowii.*

The cultured EtOAc extract of *A. sydowii* SCSIO-41301 associated with *Phakellia fusca* provided new xanthones **80** and **81**. Compound **80** is related to versicone A with 3-OH instead of the isopentene group in versicone A, while compound **81** has an additional 6-OCH_3_ group compared to compound **80** [35]. The new mycotoxin 6-methoxyl austocystin A (**83**) and the related known compound **82** were isolated from *Verrucella umbraculum*-associated *A. sydowii* SCSIO-00305 (Figure 6). Compound **83** is similar to compound **82** except for the presence of an additional C6-OCH_3_. Their 1′R/2S configuration was assigned based on X-ray analysis [24]. 

**Figure 7:**
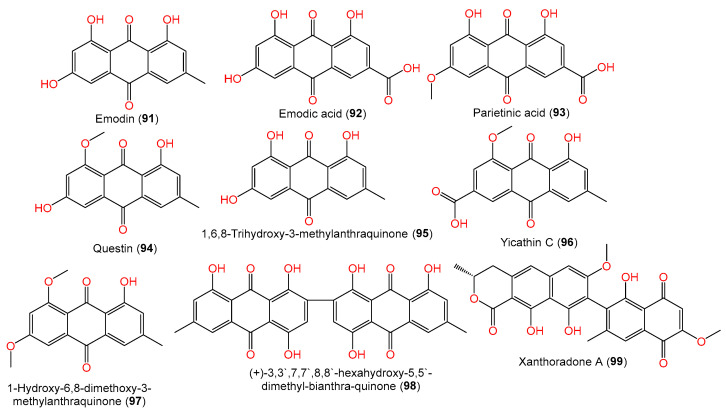
Structures of quinones (**91**–**99**) reported from *A. sydowii.*

Additionally, compounds **92**–**95** are anthraquinones reported by Liu et al. from a *Phakellia fusca*-associated fungal strain [35] (Figure 7). Compounds **98** and **99** are quinone derivatives separated from *A. sydowii* #2B associated with *Aricennia marina* by Wang et al. [64].

### 2.4. Alkaloids

Alkaloids have drawn considerable attention because of their unique structural features and varied bioactivities. Interestingly, alkaloids belonging to various classes were reported from *A. sydowii*. 

**Figure 8:**
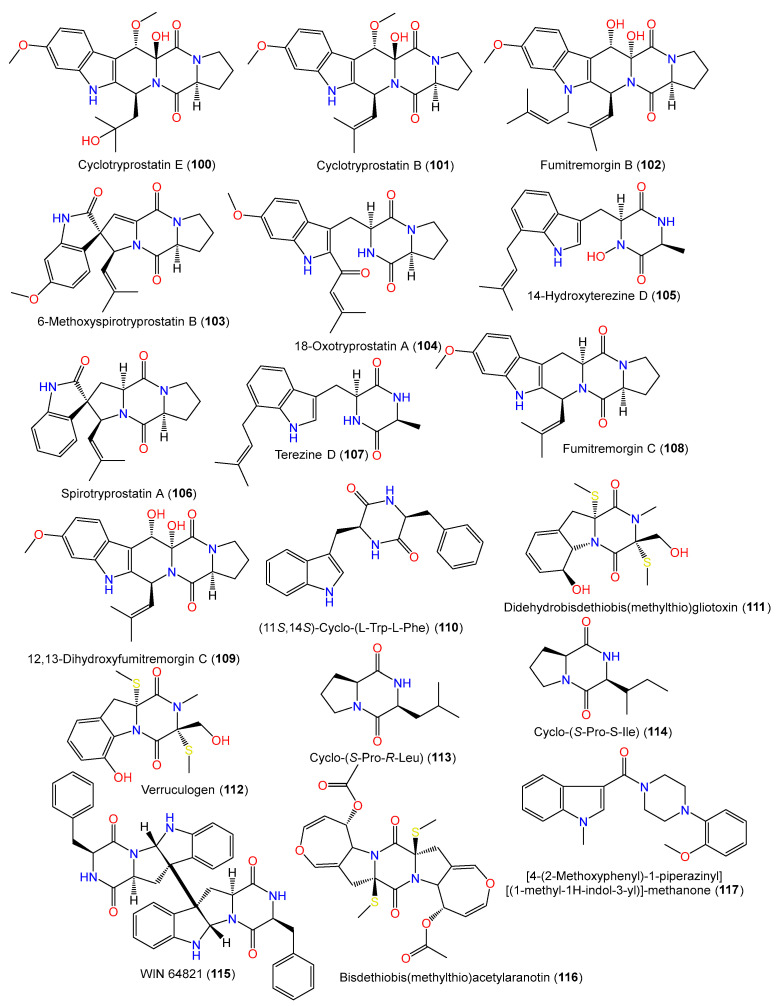
Structures of alkaloids (**100**–**117**) reported from *A. sydowii.*

From the culture broth of coral *Verrucella umbraculum*-accompanied *A. sydowii* SCSIO-00305, using bio-guided fractionation, a new indole diketopiperazine member, cyclotryprostatin E (**101**), and compounds **100**, **102**, and **117**–**123** were purified using RP-18 CC/HPLC and characterized by spectral data interpretation [31] (Figure 8). Compound **101** is similar to compound **100** bar the replacement of the tri-substituted double bond in compound **100** with an oxygen-bonded quaternary carbon; compound **117** possesses indolyl, piperazinyl, and 1,2-disubstituted phenyl groups [31].

In 2008, Zheng et al. purified new diketopiperazines **103**–**105** and a new oxaspiro [4.4]lactam-containing alkaloid, labeled **131**, along with compounds **106**–**109**, **111**, **112**, **130**, and **140**–**143** from the EtOAc extract of *A. sydowii* PFW1-13 isolated from driftwood sourced from Baishamen beach, Hainan, China, using SiO_2_/Sephadex LH-20 CC/HPLC [48]. The configurations of compounds **103**–**105** and **131** were assigned based on NMR (nuclear magnetic resonance) and CD spectral analyses, and the specific rotation was 3S/12S/18S for compound **103** and 9S/12S for compounds **104** and **105**, while compound **131** was identified as a 14-nor-derivative of compound **130** with 5*S*/8*S*/9*R*/10*S*/11*S*/12*Z* configuration [48].

**Scheme 3:**
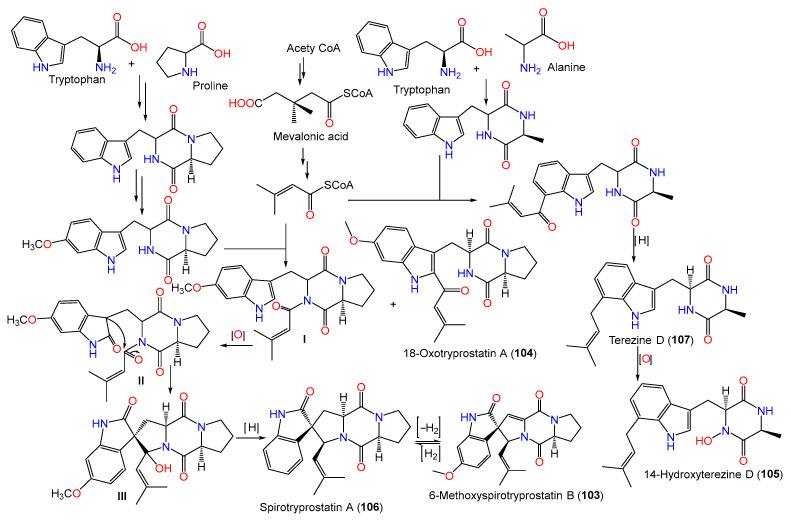
Biosynthetic pathway of compounds **103**–**106** [48].

Biosynthetically, compounds **103**–**105** were postulated to be generated through a mixed mevalonic acid/amino acid pathway. Compound **105** is generated from the oxidation of compound **107**, which results from mevalonic acid, tryptophan, and alanine. A cyclo(Trp-Pro) is formed from proline and tryptophan and is further oxidized and methylated to produce ethoxylated cyclo(Trp-Pro). Then, the latter reacts with mevalonic acid to yield compound **104** and intermediate **I**. An intramolecular aldol reaction of intermediate **I** yields intermediate **III**, which is deoxygenated to produce compound **106**. Additionally, the dehydrogenation of compound **106** gives compound **103** (Scheme 3).

Kaur et al. separated a new diketopiperazine dimer WIN 64821 (**115**) and the known compound **110** using SiO_2_ CC and RP-HPLC from the CH_3_OH/CH_3_CN extract of *A. sydowii* MSX-19583 obtained from spruce litter; the compounds were assigned by spectral and ECD analyses and Marfey’s Method (Table 4). Compound **115** is structurally similar to the ditryptophenaline reported in various *Aspergillus* species and derived from tryptophan and phenylalanine subunits [33].

**Figure 9:**
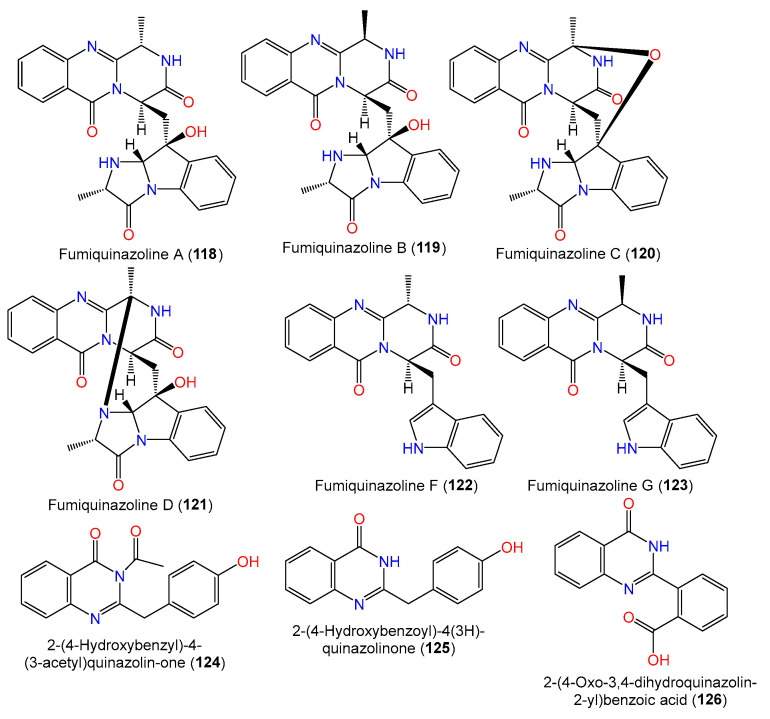
Structures of quinazoline alkaloids (**118**–**126**) reported from *A. sydowii.*

A new quinazolinone alkaloid, labeled **124**, as well as related alkaloid **125** and triazole analog **134** were separated and characterized from the mycelia EtOAc extract of seawater-derived *A. sydowii* SW9 using SiO_2_/Rp-18/Sephadex LH-20 CC and spectral analyses (Figure 9). Compound **124** is an acetyl derivative of 2-(4-hydroxybenzyl)quinazolin-4(3*H*)-one, previously reported from *Cordyceps*-associated *Isaria farinose* [41,66].

**Figure 10:**
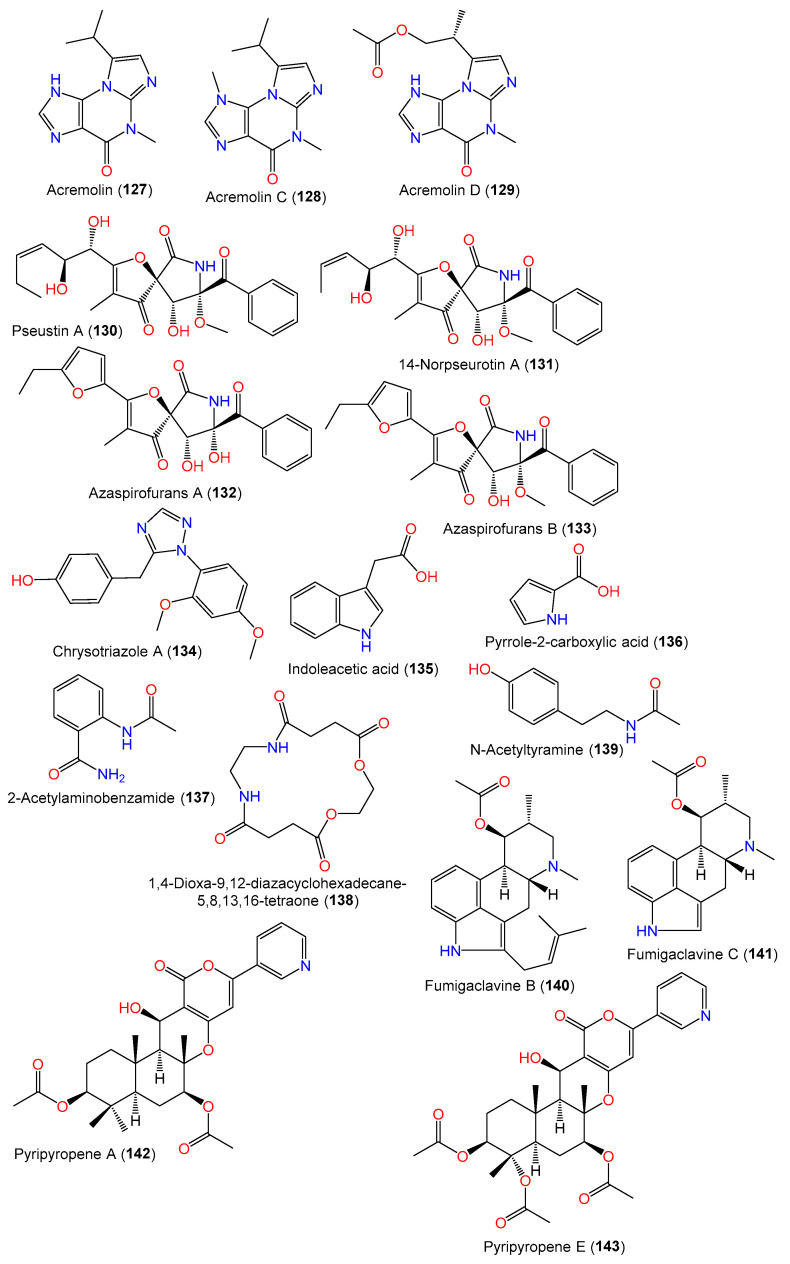
Structures of alkaloids (**127**–**143**) reported from *A. sydowii.*

Acremolins are rare alkaloids with a 5/6/5 tricyclic core, possessing an imidazole moiety fused with a methyl guanine moiety. Interestingly, acremolins were reported from *Aspergillus* species *Aspergillus* sp. S-3-75 and SCSIO-Ind09F01 and *A. sydowii* SP-1 [40,67]. From the Antarctic *A. sydowii* SP-1, a new alkaloid acremolin C (**128**) along with compound **110** were separated using SiO_2_ CC/ODS/HPLC and characterized by spectral methods. Compound **128** is a regio-isomer of acremolin B previously reported by Tian et al. from the deep-sea-derived fungus *Aspergillus sp.* SCSIO and has a isopropyl group at C-2′ instead of C-1′ (Figure 10) [40,67]. In 2022, Niu et al. purified and characterized, from the deep-sea-derived *A. sydowii* MCCC-3A00324, a new acremolin alkaloid acremolin D (**129**) along with compounds **110**, **126**, **127**, **135**, and **136** using SiO_2_ CC/HPLC and spectral and ECD data. Compound **129** is closely related to compound **127** in that one CH_3_ group in **127** has been replaced by an acetoxy methylene group [65].

New hetero-spirocyclic γ-lactam analogs azaspirofurans A (**132**) and B (**133**) were separated from the marine sediment-derived *A. sydowii* D2-6 using SiO_2_/Sephadex LH-20 CC and were characterized based on spectral and chemical evidence (Figure 10). These compounds featured an ethyl furan ring linked to 1-oxa-7-azaspiro[4,4]non2-ene-4,6-dione core [43].

### 2.5. Phenyl Ether Derivatives

Phenyl ethers are a group of simple polyketides that are widely reported in various *Aspergillus* species and have shown significant bioactivities (Table 5).

**Figure 11:**
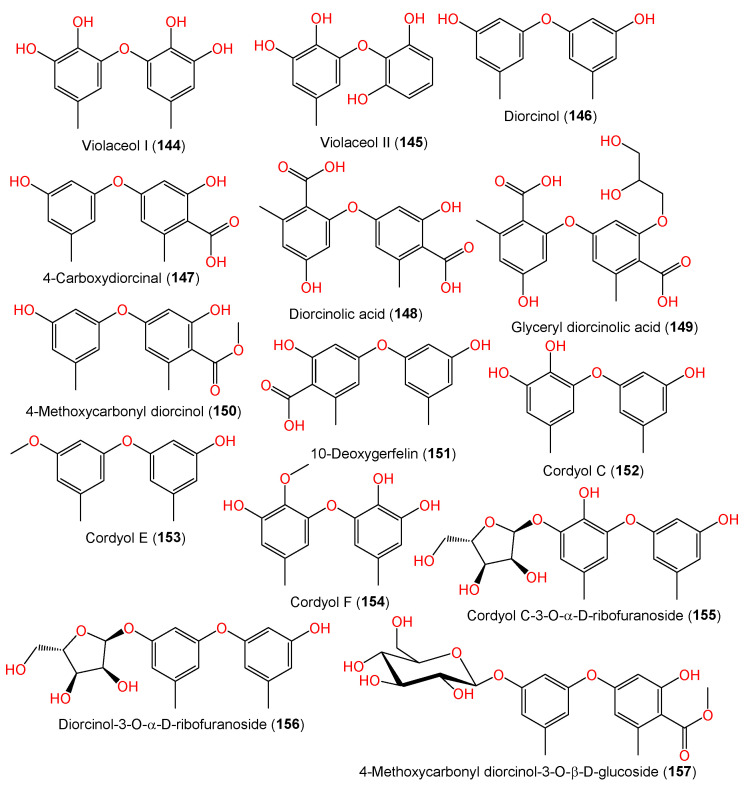
Structures of phenyl ether derivatives (**144**–**157**) reported from *A. sydowii.*

A new biphenyl ether derivative diorcinolic acid (**148**) together with compounds **144**–**147**, **152**, and **153** were separated from marine sponge *Stelletta* sp.-associated *A. sydowii* (Figure 11). Compound **149** featured two ether-linked 1,3-dioxy-6-carboxy-5-methylphenyl units. It was assigned as dicarboxylated diorcinol (carboxylated orcinol‘s ether-linked dimer) [36]. Bioassay-guided separation of the East China Sea sediment-derived *A. sydowii* MF357 yielded new tris-pyrogallol ethers sydowiols A–C (**166**–**168**) and related bis-pyrogallol ethers **144** and **145** that were characterized based on detailed spectral analysis and symmetry considerations [37]. On the other hand, the LC–UV–MS-guided separation of EtOAc extract of China Sea-derived *A. sydowii* resulted in new diphenyl ethers **155**–**157** and **159**–**161** along with compounds **146**, **147**, **149**, **150**, **152**, and **162**–**164** using SiO_2_/Sephadex LH-20 CC/HPLC; the compounds were assigned using spectral and chemical methods. Compounds **155** and **156** are rare glycosides, possessing a D-ribose moiety, whereas compound **157** has a D-glucose moiety [9].

**Figure 12:**
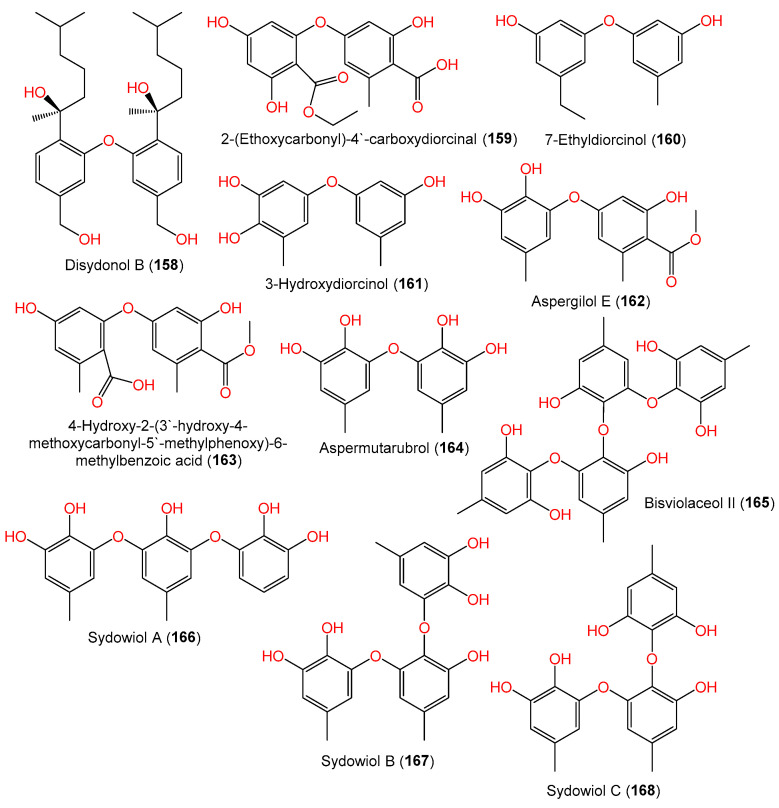
Structures of phenyl ether derivatives (**158**–**168**) reported from *A. sydowii.*

From cold seep-derived *A. sydowii* 10–31, bisviolaceol II (**165**), a new tetraphenyl ether derivative, was isolated and characterized by Liu et al. using SiO_2_/Sephadex LH-20 CC/HPLC and spectral tools, respectively [38] (Figure 12).

### 2.6. Chromane and Coumarin Derivatives

Citrinin is a polyketide-derived mycotoxin that was first reported in *Penicillium citrinum* as lemon-yellow particles. Also, other species of *Monascus*, *Penicillium*, and *Aspergillus* genera are found to be capable of producing this toxin [68].

**Figure 13:**
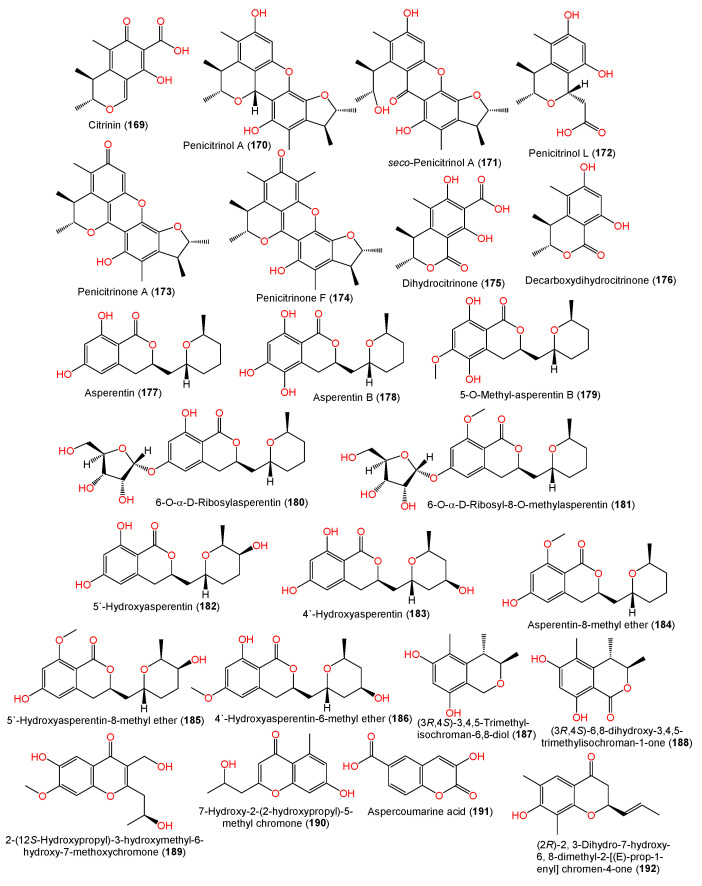
Structures of chromane and coumarin derivatives (**169**–**192**) reported from *A. sydowii.*

The coculture of two or more different microbes is a useful approach for activating silent biosynthetic genes to accumulate cryptic compounds. In this regard, an investigation on the EtOAc extract of a coculture of *A. sydowii* EN-534 and *P. citrinum* EN-535 obtained from the marine red alga *Laurencia okamurai* using SiO_2_/Sephadex LH-20/RP-18 CC/preparative TLC (thin-layer chromatography) resulted in the separation of new citrinin analogs **171** and **172**, in addition to compounds **169**, **170**, and **173**–**176**, that were characterized by spectral, optical rotation, ECD, and X-ray analyses (Table 6, Figure 13). Compounds **171** and **172** are a citrinin dimer and citrinin monomer, respectively. The configurations of compounds **171**–**173** were assigned as 3*R*/4*S*/2′*R*/3′*S*, 3*R*/4*S*/2′*R*, and 3′*S*/1*S*/3*R*/4*S* by X-ray and ECD analyses [69]. Further, asperentin B (**178**), a new asperentin analog, was obtained from the Mediterranean sea sediment-derived *A. sydowii* EN50, which is closely related to compound **177** but with an additional OH at C-6 [46]; it was proposed to be derived from the hydroxylation of PKS (polyketide synthase) precursor at the aromatic ring [46].

### 2.7. Pyrane, Cyclopentene, Cyclopropane, and Lactone Derivatives

**Figure 14:**
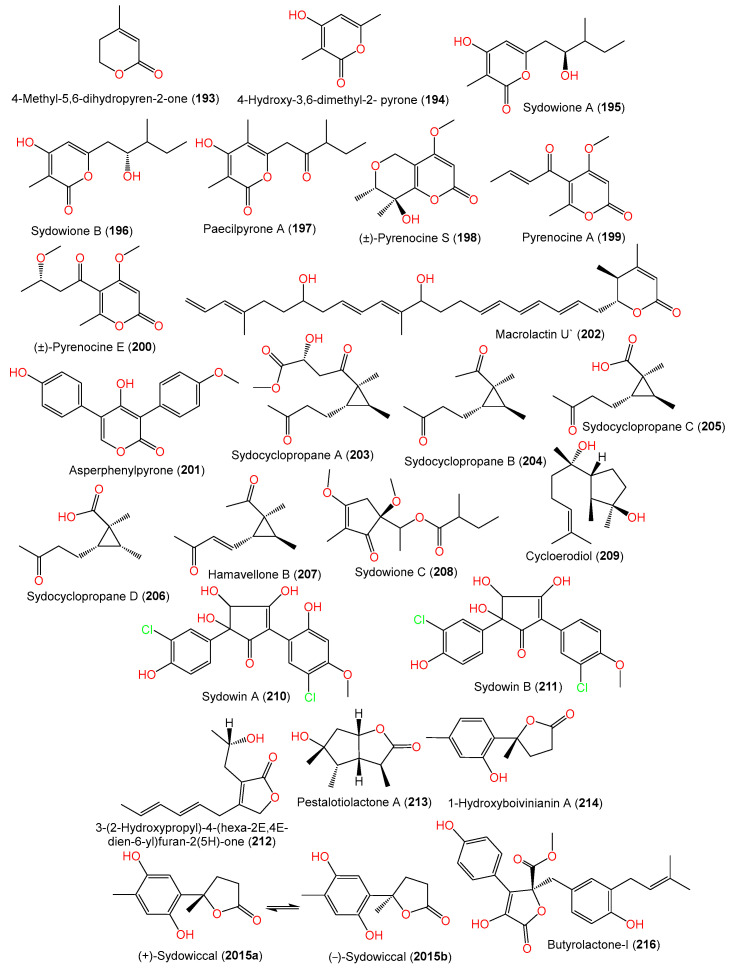
Structures of pyrane, cyclopentene, cyclopropane, and lactone derivatives (**193**–**216**) reported from *A. sydowii.*

Two new 2-pyrone derivatives **195** and **196** and a new cyclopentenone derivative **208** along with known analog **197** were isolated from the South China Sea gorgonian *Verrucella umbraculum*-derived *A. sydowii* SCSIO-00305 utilizing SiO_2_/RP-10/Sephadex LH-20 CC/HPLC (Figure 14). The 8*R*/8*S*/5*S* absolute configuration of compounds **195**, **196**, and **208** was established using Mosher’s method and ECD spectra [24]. Liu et al. separated pryan analogs **194** and **193** from *A. sydowii* SCSIO-41301 (Table 7) [35]. Two new pyrone derivatives, labeled **189** and **198**, together with compounds **199** and **200** were separated from *Aricennia marina*-inhabiting *A. sydowii* #2B by SiO_2_/Sephadex LH-20 CC/HPLC. Based on X-ray analysis and optical rotation measurement, compound **189** has 1S-configuration, while compounds **198** and **200** are racemic mixtures. Compounds **198** and **200** are alpha-pyrone derivatives; however, compound **189** is γ-pyrone [64]. Further, two undescribed α-pyrone derivatives **191** and **201** were separated and characterized from deep-sea-derived *A. sydowii* MCCC-3A00324. Compound **201** bears two phenyl moieties at C-3 and C-5 [60].

The cyclopropyl moiety is the smallest cycloalkane moiety. It is a strained moiety that usually occurs as a structural subunit of various natural metabolites, particularly alkaloids, steroids, and terpenoids [71,72]. Many polycyclic natural metabolites bearing this ring were reported in higher plants, archaea, fungi, and bacteria, while monocyclic molecules are rarely found [73]. In 1920, the first monocyclic cyclopropane (+)-trans-chrysanthemic acid was reported [42]. In 2022, sydocyclopropanes A–D (**203**–**206**), novel monocyclic cyclopropane acids, along with compound **207** were separated from the deep-sea sediment-associated *A. sydowii* MCCC-3A00324 using SiO_2_ CC/Sephadex LH-20/HPLC and were characterized by spectral, ECD, and DP4^+^ probability analyses by Niu et al. [42]. These metabolites feature a 1,1,2,3-tetrasubstituted cyclopropane moiety with different alkyl side chains. Their established configurations were 1S/2S/3S/12R for compound **203**, 1S/2S/3S for compounds **204** and **205**, and 1S/2R/3S for compound **206**, which was identified as a C-2 epimer of compound **205** [42].

In 2006, Teuscher et al. separated and characterized new hydroxylated, chlorinated diaryl cyclopentenone derivatives **210** and **211** from red alga *Acanthophora spicifera*-associated *A. sydowii* using Sephadex LH-20/HPLC and NMR/CD analyses, respectively. These kinds of metabolites were related to diaryl cyclopentenones reported in order Boletales basidiomycetic fungi and involved in conspicuous bluing reactions of fruiting bodies and reported for the first time from ascomycetes [53]. Compound **215** was isolated as enantiomers, involving (+)-(**215a**) and (−)-(**215b**), using SiO_2_/RP-18 CC/HPLC from *Rhododendron mole-*accompanied* A. sydowii* and elucidated by spectral and CD analyses. They were purified by chiral HPLC and identified to have 7*S* and 7*R* configurations, respectively [26].

### 2.8. Other Metabolites

**Figure 15:**
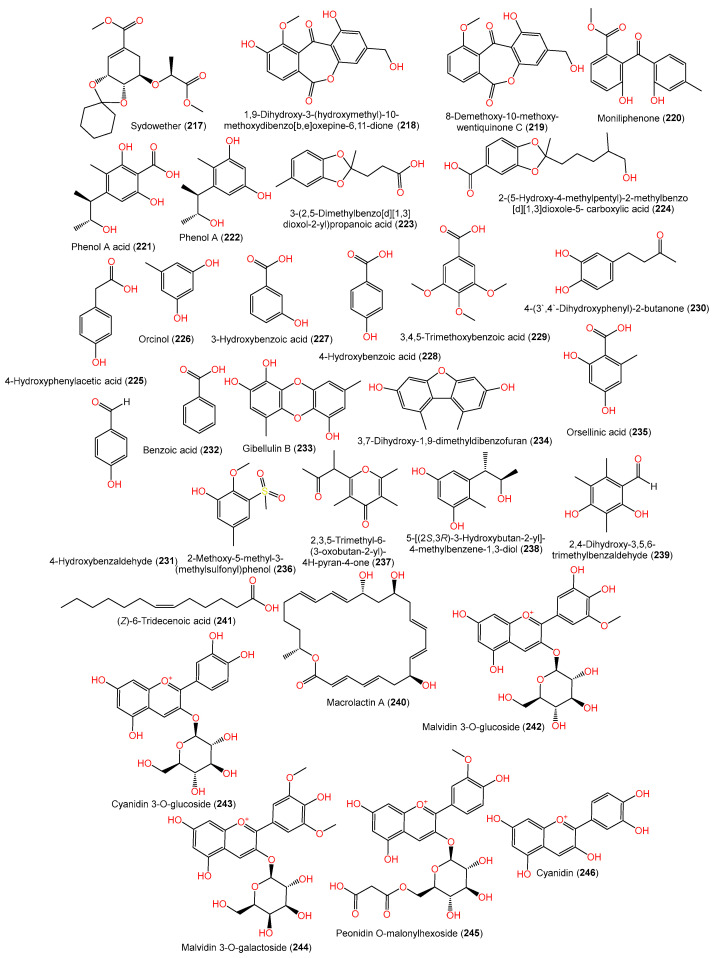
Other metabolites (**217**–**246**) reported from *A. sydowii.*

New catechol derivatives **223** and **224** were separated as racemic by Liu et al. and could not be separated into their enantiomers. Compound **224** resembles compound **223**, except for the presence of the C-2 COOH group and a 2-methylpentan-1-ol unit, instead of the 2-CH_3_ and propionic acid moiety in compound **223** [35] (Table 8, Figure 15). A new chorismic acid analog, labeled **217**, was reported by Liu et al. and its 3R/4S/5R/1′S configuration was assigned based on ECD analysis [41]. The same group separated a dibenzofuran derivative, labeled **234**, from *A. sydowii* SCSIO-41301 [35]. Compounds **228**–**230** and **234** were separated by Niu et al. from the deep-sea sediment-associated *A. sydowii* MCCC-3A00324 [60].

Anthocyanins belong to the flavonoids family and are generally reported from plant sources. These metabolites have various applications in agro-food industries such as in natural dyes; additionally, their substantial therapeutic human health in treating obesity and improving cardiovascular function are of note [74]. In 2020, Bu et al. reported the capacity of *A. sydowii* H-1 to produce anthocyanins using metabolomic and transcriptomic analyses [25]. Compounds **242**–**246** were characterized; compounds **242** and **244** were the most abundant of the identified anthocyanins [25]. Interestingly, cinnamate-4-hydroxylase and chalcone synthase genes were identified as the key genes involved in anthocyanin biosynthesis [25]. This expanded the knowledge of natural anthocyanin biosynthesis by fungi for the first time.

## 3. Biological Activities of *A. sydowii* Extracts and Its Metabolites

### 3.1. Cytotoxic Activity

*A. sydowii* MSX19583 extract (%cell viability: 54%, conc.: 20 μg/mL) had moderate cytotoxic capacity against MDA-MB-435 (human melanoma cell line) in an MTT assay [33], while a cultured EtOAc extract displayed a marked toxic effect (LD_50_ (lethal dose 50) 36 μg/mL) in a brine shrimp assay [36].

Vascular endothelial cell growth factor (VEGF) is a tumor-secreted protein that stimulates both the migration and growth of vascular endothelial cells; thus, interference with VEGF signaling suppresses tumor growth or blocks angiogenesis [34].

Compound **88** was found to suppress HUVEC (human umbilical vein endothelial cell) proliferation caused by VEGF, bFGF (basic fibroblast growth factor), or ECGS (endothelial cell growth supplement) (IC_50_s: 1.4, 2.8 μM, and 6.2 μM, respectively) compared to SU5416 (a tyrosine kinase inhibitor, IC_50_s: 0.05, 5.3, and 30.5 μM, respectively) [34] and demonstrated selective cytotoxic capacity versus A549 (human lung adenocarcinoma epithelial cell line) (IC_50_ < 10 µM) [55].

In an MTT assay, compounds **101** and **117** demonstrated a notable cytotoxic capacity toward A375 (human melanoma cell line) (IC_50_: 5.7 μM), whereas compound **101** had no cytotoxicity towards adenocarcinoma cells A549, A375, and Hela (human cervical epitheloid carcinoma cell line) compared to cis-platin [31] and compounds **1**, **45**, **110**, and **115** were inactive against MDA-MB-435 and HT-29 (human colon cancer cell lin) [33] (Table 9).

Acremolin D (**129**) had cytotoxic efficacy versus K562 (human erythroleukemic) and Hela-S3 (human cervix adenocarcinoma) cell lines with % inhibition equal to 25.1 and 30.6%, respectively, while compound **127** displayed activity (% inhibition: 20.9–35.5%) versus HepG2 (human hepatocellular liver carcinoma cell line), A549, and K562 in an MTT (3-(4,5-dimethylthiazol-2-yl)-2,5-diphenyltetrazolium bromide) assay [65]. Azaspirofurans A (**132**) displayed moderate cytotoxic potential versus A549 cell proliferation (IC_50_: 10 µM) in the MTT method [43] and compounds **103**–**105** (IC_50_s: 8.29, 1.28, and 7.31 µM, respectively) demonstrated weak capacities [48].

Compounds **44** and **149** were mildly active versus KB (human oral epidermoid carcinoma cell line), HepG2, and HCT-116 (human colon cancer cell line) cells (IC_50_s: 50–70 μM) compared to doxorubicin (IC_50_s: 5–6 μM) [36]. Wang et al. reported that compounds **146**, **149**, **152, 155**, **160**, and **161** were found to exhibit cytotoxic potential versus A549, U937 (pro-monocytic, human myeloid leukaemia cell line), HL-60 (human promyelocytic leukemia cell line), and K562 cells (IC_50_: 3.36–23.03 µM) [9]. Wang et al. stated that compounds **98**, **99**, **199**, **200**, and **216** possessed cytotoxic capacities versus VCaP (human prostate cancer cell line) (IC_50_s: 1.92–33.36 μM), but compound **189** was inactive in comparison with docetaxel (IC_50_: 4.95 nM) in the MTT method [64]. Compounds **83**, **195**, **196**, and **208** possessed toxicity towards brine shrine nauplii (LC_50_s: 2.9–19.5 µM), whereas compound **83** had a potent efficacy (LC_50_: 2.9 µM) compared to toosendanin (LC_50_: 2.2 µM) [24]. On the other hand, compounds **192** and **239** revealed powerful cytotoxic potential versus P388 (menogaril-resistant mouse leukaemia cell line) (IC_50_s: 0.14 and 0.59 µM, respectively) in a SRB (sulforhodamine B) assay; however, compound **237** was inactive [44].

### 3.2. Antioxidant and Immunosuppression Activities

Compound **24** was found to have DPPH (1,1-diphenyl-2-picrylhydrazyl) scavenging activity (IC_50_: 113.5 μM/L), while compound **25** was inactive (IC_50_ value > 300 μM/L) compared to BHT (butylated hydroxytoluene) (IC_50_: 30.8 μM/L) in a DPPH assay, suggesting that the OH position and racemization influenced the activity [32]. Also, compound **88** demonstrated antioxidant capacity (IC_50_: 17.0 μM) compared to butylated hydroxyanisole (IC_50_: 0.13 μM). Compound **88** differed from compound **71** in lacking C_7_–C_8_ double bonds, revealing that the planar structure of compound **71** might reduce its activity [56]. On the other hand, compounds **195** and **196** had more potent antioxidant activity (IC_50_s: 46.0 and 46.6 µM, respectively) than L-ascorbic acid (IC_50_: 61.0 µM); however, compounds **83** and **208** were weakly active (IC_50_s: 98.0 and 86.3 µM, respectively) [24].

Compounds **72**, **74**, **76**–**78**, **91, 94**, **97**, **218**, and **220** were evaluated in vitro for immunosuppression capacity against Con-A (concanavalin A)- and LPS (lipopolysaccharide)-induced mouse splenic lymphocyte proliferation. It was noted that compounds **91** and **94** displayed moderate potential (IC_50_s: 8.45 and 10.10 μg/mL and 10.25 and 14.10 μg/mL, respectively), compared to cyclosporin A (IC_50_: 0.62 μg/mL for Con-A and 0.53 μg/mL for LPS). Other compounds showed weak or no activity [63].

### 3.3. Anti-Mycobacterial, Anti-Microalgal, and Antimicrobial Activities

Infectious illnesses seriously threaten human health worldwide [75,76]. Recently, the increasing recurrence of pathogens’ resistance to antimicrobials represents an alarming trend in infectious diseases that results from misuse or overuse of existing antimicrobials and has become a universal health concern [75,76].

*A. sydowii* ethyl acetate extract (conc.: 500 µg/disk) had selective activity against *B. subtilis* and *E. coli* (inhibition zone diameters (IZDs) 12 and 15 mm, respectively); however, it was inactive against *S. aureus*, *C. albicans*, *Cladosporium herbarum*, and *C. cucumerinum* [53]. In another study, the EtOAc extract of *Dactylospongia* sp.-associated *A. sydowii* DC08 revealed antibacterial potential versus *E. coli* and *S. aureus* (IZDs 12.31 and 14.25 mm, respectively) [77]. Wang et al. reported that *A. sydowii* ZSDS1-F6 EtOAc extract displayed significant antimicrobial capacity versus *Aeromonas hydrophila* and *Klebsiella pneumonia* [45].

The antibacterial effectiveness of compounds **1**, **2**, **5**, **11**–**14**, **42**, **43**, and **55** versus phytopathogenic bacteria *Ralstonia solanacarum* and *Pseudomonas syringae* utilizing a broth microdilution method revealed that compound **5** had inhibition potential versus *P. syringae* (MIC (minimum inhibitory concentration): 32 µg/mL), whereas compounds **1**, **14**, and **43** were active versus *R. solanacarum* (MICs: 32 µg/mL) using the broth microdilution method [47] (Table 10). Further, compounds **11** and **14** inhibited *Fusarium oxysporum* spore germination (EC_50_s: 54.55 and 77.16 µg/mL, respectively), while compounds **1**, **11**, **14**, and **43** inhibited *Alternaria alternata* spore germination (EC_50_s: 26.02–46.15 µg/mL), suggesting the possible use of bisabolane sesquiterpenoids as anti-phytopathogens [47]. Also, compound **44** revealed antibacterial efficacy versus the human pathogen *S. aureus* and fish pathogens *S. iniae* and *V. ichthyoenteri* [36]. Compounds **71**, **88**, and **91** showed weak potential against *Vibrio rotiferianus* (MICs: 16–33 µg/mL); however, compounds **69**, **79**, **86**, **88**, **91**, and **219** were weakly active versus MRSA (methicillin-resistant *Staphylococcus aureus*) (MICs: 15–32 µg/mL) compared to erythromycin and chloramphenicol [55].

Compounds **2**, **3**, and **110** demonstrated notable antibacterial efficacy versus *S. aureus*, MRSA, *S. epidermidis*, and MRSE (MICs: 0.25–1.0 μg/mL) compared to trigecycline (MICs: 0.06–0.12 μg/mL); however, compound **128** displayed moderate-to-weak activity (MICs: 4–32 μg/mL) [40]. Compounds **42**, **50,** and **146** had moderate effectiveness versus *K. pneumonia* (MICs: 21.4, 10.7, and 21.7 μM, respectively); also, compounds **1** and **42** exhibited moderate activity against *E. faecalis* (MIC: 18.8 μM) and *A. hydrophila* (MIC: 4.3 μM), respectively, using an agar dilution method [45].

Compounds **53**, **54**, and **165** (conc.: 20 µg/disc) were found to prohibit the growth of bacteria (*V. anguillarum*, *V. harveyi*, *V. parahaemolyticus*, and *V. splendidus)* and harmful microalgae (*P. micans* and *P. minimum*) in a disc diffusion assay [38]. Pathogenic bacteria and harmful algal blooms pose substantial threats to marine aquaculture. Compounds **53** and **54** inhibited *P. micans* and *P. minimum* (IC_50_ ranging from 1.3 to 11 μg/mL), while compound **165** only had inhibitory efficacy against *P. minimum* (IC_50_: 5.2 μg/mL). Additionally, these compounds showed inhibition against *Vibrio* species (*V. anguillarum*, *V. harveyi*, *V. parahaemolyticus*, and *V. splendidus)* with IZDs ranging from 6.4 to 8.7 mm. The MICs for compounds **53** and **54** were 8 μg/mL against *V. anguillarum* and *V. parahaemolyticus* and 16 μg/mL against *V. harveyi* [38].

Compounds **61**, **62**, **130**, and **131** displayed notable growth inhibition potential versus *E. coli*, *B. subtilis*, and *M. lysoleikticus* (MICs: 3.74–87.92 µM); compounds **131** and **61** were more powerful than compounds **62** and **130** [48]. Antibacterial testing of compounds **51**, **124**, **125**, and **134** against human pathogenic bacterial strains *E. coli*, *S. aureus*, *S. pneumoniae*, and *S epidermidis* revealed that compounds **51, 124**, and **125** demonstrated selective inhibitory capacities (MICs ranging from 2.0–16 μg/mL), whereas compound **51** had significant activity against *E*. *coli* (MIC: 2.0 µg/mL) that was comparable to chloramphenicol (MIC: 2.0 µg/mL) [41].

Among the pyrogallol ethers, i.e., compounds **144**, **145**, and **166**–**168** reported by Liu et al. in 2013, compounds **166** and **168** (IC_50_: 14.0 and 24.0 μg/mL, respectively) demonstrated Mt PtpA (protein tyrosine phosphatase A) (*Mycobacterium tuberculosis* protein tyrosine phosphatase A)-inhibitory activity and compound **168** moderately inhibited *S. aureus* (MIC: 12.5 μg/mL) [37]. *M. tuberculosis* secretes PtpA into the infected macrophages’ cytosol to avoid devastation by macrophage phagocytosis. Inhibition of PtpA remarkably attenuates *M. tuberculosis* growth in human macrophages; therefore, Mt PtpA is a target for developing anti-tuberculosis drugs [37].

Liu et al. stated that compounds **144**–**146**, **152**, and **153** were moderately effective versus fish pathogens *S. iniae* FP3187, and *V. ichthyoenteri* (Vi0917-1 and Vi099-7) and human pathogen *S. aureus* (SG 511 and SG 503) [36]. Compounds **147** and **149** were inactive, suggesting that methoxy groups increased the antibacterial potential; however, the carboxyl group reduced the activity of diphenyl ether derivatives [36]. Additionally, compounds **138**, **139**, **144**, **209**, and **210** possessed moderate antibacterial effectiveness (MICs: 6.3–25.0 µM) versus series of bacterial strains [28] and compound **84** was moderately active (MICs: 64, 128, 16, 32, and 32 µg/mL, respectively) versus MRSA, MDRPA (multi-drug-resistant *Pseudomonas aeruginosa*), *E. coli*, *S. aureus*, and *P. aeruginosa* in an agar diffusion assay [39].

Compounds **137**, **169**–**172**, **174**–**176**, **221**, and **222** reported from *A. sydowii* EN-534 and *Penicillium citrinum* EN-535 coculture were examined for antibacterial potential versus strains of human and aquatic bacteria. Compounds **137**, **169**, and **170** showed antibacterial capacity against bacteria *E. coli*, *E. ictaluri*, *M. luteus*, *V. parahaemolyticus*, and *V. alginolyticus* (MICs ranged from 4 to 64 μg/mL), while compounds **137**, **171**, **172**, and **174** were active against *V. alginolyticus* and *E. ictaluri* (MICs: 32–64 μg/mL). Additionally, compound **170** had marked activity against *M. luteus* (MIC: 4 μg/mL) compared to chloramphenicol (MIC: 2 μg/mL) [69].

### 3.4. Anti-Influenza Virus Activity

The influenza pandemic remains a threat to public health because of its elevated rates of mortality and morbidity. Although vaccination is the primary means for preventing this illness, antiviral medications are an essential adjunct to vaccines for influenza control and prevention [78,79]. In the last several decades, natural products have been subjected to intensive investigations as a possible alternative therapy for the recovery and treatment of influenza. Various reports have demonstrated that developing natural bioactive metabolites has remarkable advantages [78,79]. It is noteworthy that the renowned anti-influenza oseltamivir was synthesized using natural shikimic and quinic acids as starting materials [78,79]. Some reports assessed the anti-influenza potential of *A. sydowii*-isolated metabolites; these are highlighted below (Table 11).

Interestingly, compounds **80** and **81** possessed notable selective inhibition versus two influenza A virus subtypes, including A/Puerto Rico/8/34 (H1N1) and A/FM-1/1/47 (H1N1) (IC_50_s: 2.17–4.70 μM), compared to ribavirin (IC_50_s: 2.53 to 6.23 μM). Additionally, compounds **92** and **94** had potent efficacy on A/Puerto Rico/8/34 (H1N1) (IC_50_s: 1.92 and 2.0 μM, respectively). Furthermore, compound **234** demonstrated broad inhibitory potential against A/Puerto Rico/8/34 (H1N1), A/Aichi/2/68 (H3N2), and A/FM-1/1/47 (H1N1) (IC_50_s: 1.31, 1.24, and 2.84 μM, respectively) compared to ribavirin (IC_50_s: 2.53, 6.23, and 3.97 μM, respectively) [35]. Compounds **50**, **146,** and **152** demonstrated weak anti-H3N2 potential (IC_50_s: 57.4, 66.5, and 78.5 μM, respectively) in a CPE (cytopathic effect) inhibition assay compared to Tamiflu (IC_50_: 0.95 μM) [45]. Further, Yang et al. stated that compounds **137**, **169**–**172**, and **174** demonstrated anti-influenza NA (neuraminidase) activity, with compounds **137** and **174** displaying better efficacy (IC_50_s: 12.9 nM and 18.5 nM, respectively) compared to oseltamivir (IC_50_: 3.6 nM) [69]. Additionally, compounds **203**–**207** demonstrated antiviral potential versus the A/WSN/33 virus (H1N1) (IC_50_s ranged from 26.7 to 77.2 μM), compared to oseltamivir (IC_50_: 18.1 μM); compounds **203**, **204**, and **207** were the most active (IC_50_s: 26.7, 29.5, and 35.8 µM, respectively). It was found that the C-1 methyl 2-hydroxy-4-oxobutanoate side chain significantly enhanced the antiviral activity (e.g., compound **203** vs. compound **205**) and C-3 configuration had less influence on activity (e.g., compound **205** vs. compound **206**) [42].

### 3.5. Anti-Diabetic and Anti-Obesity Activities

A close relation among between diabetes and obesity has been proven [80]. Insulin-triggered cellular glucose uptake is a crucial step in glucose regulation and any defect in this mechanism results in insulin resistance [81]. Enhancement of insulin sensitivity is one of the significant hallmarks of anti-diabetic agents. Lipid accumulation in diabetic patients can result in serious effects such as diabetic cardiomyopathy [82]. Hence, efficient anti-diabetics should decrease adipocytes’ lipid accumulation and facilitate lipid metabolism and burning [54].

In an anti-diabetic assay, compounds **1**, **5**, **42, 45**, **46**, **47**, **49**, **69**, **71**, and **88** were found to increase differentiated 3T3-L1 (fibroblast embryo mouse cell line) adipocytes’ medium glucose consumption. Among them, compound **45** significantly reduced culture medium glucose concentration (324.6 mg/dL) by 24% compared to control (glucose: 427.4 mg/dL). It was noted that the presence of a methylene alcohol and a hydroxy group on C-3 and C-7, respectively, in bisabolane sesquiterpenes is substantial in promoting 3T3-L1 adipocytes’ glucose uptake [54]. Additionally, their efficacy on differentiated 3T3-L1 adipocytes’ lipid accumulation utilizing oil-red O stain revealed that compound **45** notably prohibited lipid accumulation up to 48% in a 3T3-L1 adipocyte culture medium, indicating the compound **45** promoted glucose consumption and suppressed lipid accumulation in adipocytes [54].

### 3.6. Protein Tyrosine Phosphatase Inhibition

Protein tyrosine phosphatases (PTPs) are proven to be substantial new targets for new anti-diabetes [58]. For example, PTP1B (protein tyrosine phosphatase 1B) negatively regulates insulin action in the insulin receptor signaling pathway, SHP1 (SH2-containing protein tyrosine phosphatase 1) negatively controls signaling pathways, which streamlines glucose homeostasis through modulating insulin signaling in muscles and the liver, and CD45 (leukocyte common antigen) is a receptor for some ligands and regulates SHP-1 recruitment [58]. Also, PTP1B has a substantial role in cancer development, inflammation processes, and insulin signaling cascade. Therefore, PTP1B inhibitors are considered drug candidates for treating cancer, diabetes, inflammation processes, and sleeping sickness [46].

Asperentin B (**178**) had potent PTP1B inhibition capacity (IC_50_: 2.05 μM) compared to suramin (IC_50_: 11.85 μM). It was sixfold more potent than suramin, suggesting its possible application in anti-diabetes and anti-sleeping sickness therapeutic agents [46]. Furthermore, compounds **1**, **3**, and **18** displayed significant PTP1B-inhibitory potential (IC_50_s: 7.97, 15.88, and 14.18 μM, respectively), while compounds **1**, **2**, **18**, and **240** had potent activity towards SHP1 (IC_50_s: 8.35, 15.72, 11.68, and 14.61 μM, respectively). The PTP1B data indicated that the side chains influenced activities [58].

### 3.7. Anti-Inflammation Activity

Compounds **42**, **45**, and **88** markedly inhibited fMLP (tripeptide N-formyl-L-methionyl-L-leucyl-L-phenylalanine)/CB (cytochalasin B)-caused superoxide anion generation (IC_50_s: 5.23, 6.11, and 6.00 µM, respectively) and elastase release (IC_50_s: 16.39, 8.80, and 6.60 µM, respectively) by neutrophils [54]. It is noteworthy that compounds **1**, **5**, **46**, and **49** had selective inhibition versus fMLP/CB-caused superoxide anion generation [54]. These results demonstrated the importance of C-7 OH (compound **45** vs. compound **46**) and C-3 methylene alcohol (compounds **46**, **45**, and **49** vs. compounds **1** and **5**) on activity (Table 12). On the other hand, compound **71** also revealed a significant superoxide anion generation inhibition capacity (IC_50_: 21.20 µM) compared to compound **69** [54]. The isolated metabolites, compounds **1**–**3**, **26**–**42**, **45**, **47**, **50**, **56**–**58**, and **214**, showed a dose-dependent inhibition of LPS-induced NO (nitric oxide) secretion (conc.: 10 and 5 µM) in BV-2 microglia cells using a CCK-8 (cell counting kit-8) assay. Compounds **33**, **39**, **42**, **47**, **50**, and **57** revealed an inhibition rate >45% (conc.: 10 µM). The structure–activity relation indicated that the Δ^7,8^ double bond in sydowic acid derivatives enhanced NO secretion inhibition (e.g., compound **33** vs. compound **26**). Compound **39**, with a 56.8% inhibition rate, was found to exert its anti-inflammation activity by prohibiting the NF-κB (nuclear factor kappa B)-activated pathway [57].

It was found that compounds **145** and **153** mildly suppressed NO production induced by LPS-NO in RAW 264.7 cells (IC_50_: 73 μM) compared to dexamethasone (IC_50_: 18 μM) [36]. Additionally, compounds **59**, **60**, **146**, **152**, **191**, **201**, **213**, **228**–**230**, and **234** demonstrated an inhibitory capacity of NO production induced by LPS in BV-2 microglia cells without toxicity according to a CCK-8 assay. Interestingly, compound **234** (10 µM) was the most potent (inhibition rate: 94.4%) among these tested compounds (inhibition rate: 10.2–35.4%) [60].

Compounds **98**, **189**, **199**, and **200** possessed inhibitory effectiveness on LPS-boosted NO production in RAW264.7 cells (IC_50_s: 25.25–43.08 μM), compared to dexamethasone (IC_50_: 35.17 uM) [64]. Recently, Chen et al. reported that compounds **215** and **236** exhibited weak inhibition of LPS-induced NO production (20.1, 21.5, and 18.1%, respectively), compared to dexamethasone (% inhibition: 99.9%) in RAW 264.7 cells using a Griess reaction assay [26].

### 3.8. Anti-Nematode Activity

Globally, parasitic nematodes cause diseases of major socio-economic significance to humans and animals. They have a long-term impact on human health, especially in children [83]. Indeed, nematodes’ resistances to available anti-nematode agents are widespread all over the world [84]. Thus, there is an insistent demand to discover new agents for the effective and sustained control of nematodes.

Sun et al. evaluated the anti-nematode activity of compounds **1**–**3**, **18**–**21**, **202, 235**, and **240**. It is noteworthy that only compound **3** showed anti-nematode potential (IC_50_: 50 μM) [58]. A study by Yang et al. revealed that compounds **1**, **11**, and **14** possessed nematicidal potential versus second-stage juvenile *Meloidogyne incognita* (J2s); compound **1** had the strongest activity (% mortality: 80% at 60 and LC_50_: 192.40 µg/mL). Furthermore, compounds **1, 11**, and **14** paralyzed the nematode and then impaired its pathogenicity [47].

## 4. Industrial and Biotechnological Applications

The discovery and development of effective enzymes for the use of renewable resources as raw materials is a requirement for the transition to a biobased economy. Many enzymes are crucial in efficiently hydrolyzing raw materials by enzymatic means. Exploring the potential of untapped natural habitats is a potent method for overcoming the limited enzymatic toolkit.

*A. sydowii* was found to be a rich source of enzymes with marked industrial and biotechnological potential, including α-amylases, lipases, xylanases, cellulases, keratinase, and tannases, which are discussed here.

### 4.1. α-Amylase, Tannases, and Lipase Enzymes

Amylases (AAs) are utilized in multiple manufacturing processes, including fermentation, textile, detergent, paper, and pharmaceutical sectors [85]. Given the low cost and wide availability of the starch feedstock used to make food, bioethanol, textile, paper, detergent, and chemicals, there is a significant demand for α-amylase [86]. However, because of advancements in biotechnology, the use of AAs has increased in a variety of sectors such as those of clinical, pharmaceutical, and analytical chemistry, as well as in the food, textile, and brewing industries [85]. The huge industrial demand for AAs to support economically competitive manufacturing processes is still being severely hampered by the cost and effectiveness of AA cocktails [19]. In this regard, it is imperative to generate effective and affordable AAs by using inexpensive sources such as agricultural wastes.

Adegoke and Odibo produced AAs from *A. sydowii* IMI-502692 utilizing the solid-state fermentation of buffered cassava root fiber. It was found that this activity was enhanced by Ca^2+^, Cu^2+^, and Zn^2+^; however, it was prohibited by Fe^2+^, Sr^2+^, Ni^2+^, and Mn^2+^ [19].

A study by Elwan et al. reported that *A. sydowii* had a potential for lipase production (lipase yield of 90 µ/mL) in optimum culture conditions, specifically 5.4 pH; 2.0% sucrose, 0.2% corn oil, 0.23% (NH_4_)_2_SO_4_, 0.1% KH_2_PO_4_, 0.05% MgSO_4_·7H_2_O, 0.05%KCl, and using 0.1 M phosphate–citrate buffer and incubating at 30°C for 20 h [22].

Tannase, an extracellular enzyme belonging to the hydrolase family, is derived from various species of the *Aspergillus* genus [8,87]. It catalyzes the breakdown of depsides and tannins. Tannase lessens tannins’ unwanted effects (astringent and bitter taste), enhancing the flavor qualities of products such as animal feeds and foodstuffs. It is used in various applications, including polyphenolic compound structural elucidation, bioremediating tannin-contaminated wastewaters, gallic acid production, and coffee-flavored soft drink, fruit juice, and instant tea production [20].

In 2020, Albuquerque et al. purified and characterized tannase-acyl hydrolase from *A. sydowii* SIS-25 derived from Caatinga soil (Serra Talhada, Pernambuco, Brazil) utilizing a polyethylene glycol-citrate aqueous two-phase system. This enzyme removed phenolic components and enhanced the sensory qualities of green tea and produced gallic acid [20].

### 4.2. Bioremediation and Biodegradation

Sustainable development goals (SDGs) target various concerns in our planet such as food security, health, environmental sustainability, bioremediation, climate change, alternative eco-friendly fuel, improving water quality, sustainable food production, and discovering new drugs [88]. Treatment and measurement of various contaminants in water, soil, and air are complicated issues and are linked to the nature of contaminants and their environmental interactions. Reusing wastewater offers a substitute supply for the irrigation of agricultural land that has been used for decades in many nations. Recycling wastewater adheres to circular economy principles by reducing waste and encouraging ongoing resource reuse [89] which potentially assists various national initiatives in promoting sustainable agriculture methods. Creating agricultural systems with minimal required inputs and zero waste contributes to SDG 2 (End hunger) (via sustainable food production), SDG 12 (Responsible consumption and production), SDG 13 (Climate action), and SDG 15 (Sustainable use of terrestrial ecosystems) [90]. Various researches have focused on biologically based methods, relying on natural processes to remove contaminants such as the utilization of microorganisms (bioremediation) such as fungi to remarkably contribute to achieving the SDGs [88].

#### 4.2.1. Polycyclic Aromatic Hydrocarbons

PAHs (polycyclic aromatic hydrocarbons) are a heterogeneous class of hydrocarbons having two or more fused aromatic rings. In nature, they are formed as a result of organic matter’s incomplete decomposition and human activities such as petroleum spilling, waste incineration, home heaters, and the burning of carbon, oil, gas, or wood [91]. Additionally, PhCs (pharmaceutical compounds), a second class of contaminants, have become more significant in recent years as a result of their durability and abundance in surface water bodies and the ineffectiveness of treatment facilities eliminating them [92]. According to Olicón-Hernández et al., these contaminants are hazardous to aquatic life and contribute to microbial resistance’s emergence [93]. Numerous studies have focused on the microbial biodegradation of these contaminants, particularly by fungi [93,94], because these pollutants are known for their high toxicity and persistence [94]. It is noteworthy that halophilic fungi are useful in xenobiotic mycoremediation under high-salinity conditions [94].

González-Abradelo et al. studied the potential of *A. sydowii* EXF-12860 toward the bioremediation of saline wastewaters, containing toxic and persistent PAHs and PhCs. It was stated that *A. sydowii* may be helpful in lowering the amounts of harmful PAHs and PhCs under high-salinity conditions (>1 M NaCl) during the biotechnological downstream processing of diverse industrial wastewater. It removed 100% of fifteen complex PAHs at 500 ppm in biorefinery wastewater at high salt concentrations. Additionally, it has ecotoxic activity as it demonstrated the same capability to eliminate PhCs. This supported its capabilities for xenobiotic biodegradation in low-water activity [94]. A novel piezo-tolerant and hydrocarbon-oclastic deep-sea sediment-derived *A. sydowii* BOBA1 demonstrated a marked degradation potential for PAHs in spent engine oil hydrocarbon fractions (71.2 and 82.5% of spent engine oil, respectively) under high-pressure (0.1 and 10 MPa, respectively) culture conditions with a 21-day retention period. This provided insights into the bioremediation of hydrocarbon-contaminated deep-sea environments [95].

Additionally, Birolli et al. stated that *A. sydowii* CBMAI-935 isolated from a non-contaminated site on the coast of São Sebastião (Brazil) biodegraded anthracene [96]. To biodegrade dieldrin, one of the most widely employed organo-chlorine pesticides, banned due to its long persistence and high toxicity to the environment, Birolli et al. found that *A. sydowii* CBMAI-935 and *A. sydowii* CBMAI-933 were capable of growing in the presence of dieldrin, suggesting its high tolerance. It is noteworthy that no biodegradation byproducts were found in the GCMS, revealing that dieldrin could be converted into polar molecules or mineralized, prohibiting the emergence of harmful or durable derivatives [97].

#### 4.2.2. Heavy Metals and Insecticides

Cadmium (Cd) is often used in the electroplating and metallurgical industries and is found in several pesticides, fertilizers, and fungicides [98]. Upon its absorption by both animals and humans, it accumulates in the kidneys and liver, severely harming the renal tubules and resulting in a variety of symptoms such as proteinuria and hyperglycemia [99]. Trichlorfon (TCF) is a broad-spectrum organic phosphorus pesticide that is utilized for controlling pests on a variety of crops [100]. It is an inhibitor of cholinesterase that causes delayed neuropathy in both animals’ and humans‘ nervous systems [98].

Zhang et al. reported that by inoculating *A. sydowii* into Cd-TCF co-contaminated soil, TCF breakdown was accelerated, and soil enzyme activity was raised. When *Brassica juncea* (Indian mustard) was planted along with *A. sydowii* inoculation, maximum TCF degradation and Cd removal efficacy were noted. *Brassica juncea* is among those hyperaccumulator plant species that are frequently employed for heavy metal phytoextraction from contaminated soil. Thus, using *B. juncea* and *A. sydowii* together is a promising strategy to bioremediate soil that has been contaminated with both TCF and Cd [98]. Tian et al. isolated PAF-2, a new strain of *A. sydowii* from pesticide-contaminated soils, that had potential for the biodegradation of TCF and its degradation [100].

Esfenvalerate (S,S-fenvalerate), is a pyrethroid insecticide that deposits in marine sediments and is extremely harmful to aquatic creatures. Birolli et al. examined its biodegradation by marine-associated *A. sydowii* CBMAI-935. This strain metabolized esfenvalerate into 3-phenoxybenzoic acid, 2-(4-chlorophenyl)-3-methylbutyric acid, and its dihydroxylated derivatives [101].

Alvarenga et al. assessed the biodegradation of a commercial formulation of chlorpyrifos (Lorsban 480 BR), which is one of the most widely utilized organophosphate pesticides, by marine-derived *A. sydowii* CBMAI-935 associated with *C. erecta*. The fungus degraded ≈ 63% of the chlorpyrifos and decreased the concentration of its hydrolysis product 3,5,6-trichloropyridin-2-ol after 30 days [102]. In 2021, Soares et al. reported that this fungus also metabolized chlorpyrifos and profenofos to 3,5,6-trichloro-1-methylpyridin-2(1H)-one/2,3,5-trichloro-6-methoxypyridine/tetraethyl dithiodiphosphate/3,5,6-trichloropyridin-2-ol and 4-bromo-2-chlorophenol/4-bromo-2-chloro-1-methoxybenzene/O,O-diethyl S-propylphosphorothioate, respectively [103].

Methyl parathion is an efficient organophosphate acaricide and insecticide that is widely utilized for pest control on a wide variety of crops, but it is extremely toxic. Alvarenga et al. reported the ability of *A. sydowii* CBMAI-935 to biodegrade this pesticide completely after 20 days. This fungus metabolized this pesticide to its more toxic isomerization and oxidation products isoparathion and methyl paraoxon, which were subsequently metabolized to the less toxic product 1-methoxy-4-nitrobenzene/p-nitrophenol/O,O,O-trimethyl phosphorothioate/O,O,S-trimethyl phosphorothioate/trimethyl phosphate, suggesting *A. sydowii* CBMAI-935’s efficiency in the bioremediation of this pesticide and its toxic forms [103,104].

#### 4.2.3. Lignocellulosic Biomasses

Due to the acute energy crisis and increased demand for fossil fuels, lignocellulose is widely considered a potential cost-effective, renewable resource for bioethanol production [105,106]. Lignocellulose consists of cellulose, hemicellulose, and lignin. Lignin, which together with hemicellulose and cellulose makes up the majority of a plant’s skeleton, is the second-most abundant organic renewable resource on Earth after cellulose [105,106]. The ligninolytic enzymes Lac (laccase), LiP (lignin peroxidase), VP (versatile peroxidase), and Mnp (manganese peroxidase) play a major role in the breakdown of lignin [105,106] and are found among the extracellular enzymes in filamentous fungi. These enzymes play a significant role in bioremediation, as they neutralize or degrade contaminants in the environment [6]. They also have a wide range of uses in the paper, textile, cosmetic, food, chemical, agricultural, and energy industries.

A thermostable, low-molecular-weight xylanase belonging to the glycosyl hydrolase 11 family was purified from *A. sydowii* MG49 by Ghosh et al. and demonstrated specific efficacy only in the presence of xylan and had no activity in the presence of cellulose or carboxymethyl cellulose [23].

*A. sydowii* MS-19 isolated from the Antarctic region produced low-temperature lignin-degrading enzymes LiP and Mnp. These results suggested that *A. sydowii* MS-19 could be used as a source of lignocellulosic enzymes [107].

Xylan is the prime constituent of hemicellulose. Its backbone consists of a linear chain of 1,4-linked β-D-xylopyranosyl units, which are substituted with α-L-arabinofuranosyl, 4-O-methyl-α-D-glucuronopyranosyl, or acetyl units. It is degraded by β-D-xylosidases, endo-1,4-β-xylanases, α-glucuronidases, α-l-arabinofuranosidases, acetyl xylan esterases, and ferulic acid esterases [108].

Brandt et al. stated that *A. sydowii* Fsh102 isolated from shrimp shells showed notable xylanase-producing capacity [109]. Two xylanases I and II belonging to GH-11 (glycoside hydrolases) and GH-10 families, respectively, were characterized and expressed in *E. coli.* These enzymes can function in a wide pH range and are tolerant of mesophilic temperatures. Both xylanases can be characterized as being extremely interesting for the enzymatic breakdown of xylan-containing biomasses in industrial bioprocesses based on their activity and stability [109]. In another study on *A. sydowii* SBS-45 culture filtrate, two xylanases (I and II) were purified. They showed optimum activity at 50 °C and 10.0 pH. This activity was boosted by certain metal ions and L-tryptophan [110].

Cellulose breakdown is carried out by cellulases, including β-glucosidase, endoglucanase, and cellobiohydrolase [108,111,112]. Cellulase has wide applications in various fields like oil extraction, agricultural industries, food processing, waste management, carotenoid extraction, animal feed, brewery, textile, bio-stoning, color clarification, paper, laundry, pulp, detergent industry, and deinking [108,111,112].

*A. sydowii* isolated from Indore, India, had the potential to produce cellulases under submerged fermentation. It was found that β-glucosidase, exoglucanase, and endoglucanase were produced at a ratio of 64:27:9, whereas lactose was the best carbon source for inducing cellulase production [113].

#### 4.2.4. Keratinous Wastes

Keratins are components of hooves, wool, horns, nails, hair, and feathers [8,114]. They are insoluble proteins with highly stable polypeptide chains, containing many disulfide bonds [115,116]. According to estimates, the United States, China, and Brazil produce 40 million tons of keratinous waste each year [117]. Also, keratinous waste is produced in millions of tons annually in meat industry slaughterhouses worldwide [115,116]. Normal enzymes such as papain and pepsin that break down proteins cannot break them down. Keratinous waste management utilizing a low-cost solution is needed particularly in underdeveloped nations. These wastes can be broken down by microbial keratinases which are extracellular enzymes secreted by various bacterial and fungal genera [8,114]. They are widely used in different pharmaceutical industries, in treating keratinized skin, calluses, acne, and psoriasis, and in cosmetic products manufacture (e.g., nutritional lotions, anti-dandruff shampoos, and creams) [21,115,116]. Also, they are usually employed in nitrogen fertilizers, feed formulas, and the leather industry, as well as in treating keratin waste-contaminated wastewater [21].

Alwakeel et al. studied the capability of keratinase produced by *A. sydowii* AUMC-10935 isolated from male scalp hair to degrade keratinous materials from chicken feathers. The enzyme had optimal activity (120 IU/mg) at 50 °C and pH 8.0, which was notably prohibited by EDTA and certain metal ions [21].

### 4.3. Biocatalysis

The pharmaceutical sector is continually looking for new approaches to new therapeutic agent syntheses, which has increased the demand for biocatalytic techniques [118]. Whole microorganism cells are effectively used as catalysts in the stereoselective biotransformation of a variety of chemical molecules. Also, many chemical reactions such as carbonyl ketone reduction, sulfide oxidation, secondary alcohol deracemization, and Baeyer–Villiger reactions were all catalyzed by enzymes from various microorganisms [6]. The whole cell of *A. sydowii* was investigated as a biocatalyst for various chemical reactions. This was highlighted in the current work.

Whole cells of the marine sponge-derived *A. sydowii* Gc12 obtained from the South Atlantic Ocean catalyzed the hydrolysis of (R,S)-benzyl glycidyl ether to produce (R)-benzyl glycidyl ether. Derivatives of glycidyl ether are potentially beneficial intermediates in the manufacture of β-adrenergic blockers. *A. sydowii* Gc12 hydrolases showed regioselectivity in opening the epoxide ring of racemic oxirane [119].

Sponge-associated *A. sydowii* CBMAI-934 derived from *Chelonaplysilla erecta* produced oxidoreductase that catalyzed regioselective mono-hydroxylation of (−)-ambrox^®^ to 1β-hydroxy-ambrox. (−)-Ambrox^®^, a naturally occurring terpene, was separated from ambergris, a pathological substance formed in the blue whale’s intestine. This compound is of great commercial value in the perfume industry as a fixative or fragrant agent [120]. de Paula and Porto investigated progesterone biotransformation by *A. sydowii* CBMAI-935 associated with marine sponge *Geodia corticostylifera*. In a good yield, this fungus was able to oxidize progesterone at the C17-site, resulting in the two major products testololactone and testosterone. Additionally, this Baeyer–Villiger reaction-based bio-oxidation revealed the existence of crucial enzymes in this fungus that can aid in related steroid biotransformation [121]. *A. sydowii* CBMAI-935 only produced 2′,4-dihydroxy-dihydrochalcone with a yield of 26% from 2′,4-dihydroxy-dihydrochalcone [122].

Further research was conducted by de Oliveira et al. to assess the potential of *A. sydowii* CBMAI-934 isolated from the marine sponge *Chelonaplysilla erecta* in converting a number of methylphenylacetonitriles into corresponding acids at a high yield. It was found that aryl aliphatic nitrilases were induced by phenyl acetonitrile. Thus, *A. sydowii* CBMAI-934 might serve as a biocatalyst for the production of carboxylic acids from nitriles [123]. Zhou et al. reported that *A. sydowii* PT-2 isolated from Pu-erh tea degraded theobromine to 3-methylxanthine in a liquid culture through N-7 demethylation [124]. Also, Jimenez et al. reported that *A. sydowii* CBMAI 935 associated with *C. erecta* sponge collected from Sao Sebastiao, São Paulo, Brazil, enantioselectively reduced ene of E-2-cyano-3-(furan-2-yl)acrylamide to (R)-2-cyano-3-(furan-2-yl)propanamide with a high yield [125]. In 2018, Morais et al. studied the reduction of α-chloroacetophenones to (S)-alcohols using whole cells of marine-derived *A. sydowii* CBMAI 935 [126]. α-bromoacetophenones‘ biotransformation by the marine-derived *A. sydowii* Ce19 was studied by Rocha et al. in 2010 [127]. This fungus accelerated α-bromoacetophenones’ bioconversion into (R)-2-bromo-1-phenylethanol (56%), in addition to acetophenone (4%), 1-phenylethan-1,2-diol (26%), phenylethanol (5%) and α-chlorohydrin (9%). The substituted p-nitro- and p-bromoacetophenone’s biotransformation produced a low-concentration complex combination of breakdown products [127]. In 2017, Alvarenga and Porto tested the biocatalytic ability of *A. sydowii* CBMAI-935 of marine origin to convert 2-azido-1-phenylethanone and some derivatives to related alcohols for use in the synthesis of enantiomerically bioactive β-hydroxy-1,2,3-triazoles. *A. sydowii* CBMAI 935 displayed extremely high stereoselectivity and conversion values for the bio-reduction of 2-azido-1-phenylethanones to (S)-2-azido-1-phenylethanols [128]. Further, the marine-derived *A. sydowii* Ce15 converted 1-(4-methoxyphenyl)ethenone to (R)-1-(4-methoxyphenyl)ethanol [129].

## 5. Nanoparticle Synthesis

Nanoparticles (NP) have attracted great interest recently because of their apparent applications in different fields such as biosensors, biomedicine, cosmetics, drugs, photocatalysis, animal dietary supplements, biolabeling, etc. [130]. Conventional NP synthesis approaches are not environment-friendly and are cost-intensive. Therefore, the development of biocompatible, environment-friendly, and non-toxic protocols in nanostructure biosynthesis is a wealthy area for scientific research, wherein the use of microbes could be an auspicious alternative [131,132]. Fungi are more effective organisms for these purposes than other microbes because of their special features, including their greater growth capacity, greater potential to produce a variety of enzymes, richness in mycelial branching, ability to accumulate different metals, and capacity to grow in harsh environments [133].

*A. sydowii* derived from Bhavnagar coast water (Gulf of Khambhat, India) had a remarkable intra/extracellular capacity to biosynthesize gold nanoparticles with variable sizes depending on gold ion concentration [52]. Additionally, silver NPs were biosynthesized by Wang et al. using soil-derived *A. sydowii* culture supernatants. These NPs revealed an in vitro antiproliferative capacity against MCF-7 (human breast adenocarcinoma cell line) and HeLa cells and efficient antifungal potential versus various clinical pathogenic fungi [134].

Zhang et al. prepared magnetic chitosan microsphere-immobilized *A. sydowii* by utilizing the cross-linking of γ-Fe_2_O_3_ magnetic chitosan nanocomposites with *A. sydowii* through the instant gelation method. This microsphere demonstrated marked Cu adsorption capacity (19.21 mg/g) and good regeneration properties after four cycles, suggesting its potential application as a biosorbent for treating heavy metal-contaminated water [51].

The AgNPs synthesized by Nayak and Anitha from dune-associated *A. sydowii* had significant antimicrobial potential versus selected bacterial stains; its combination with vancomycin and ampicillin showed enhanced activity (by sevenfold against *Shigella* sp. and by sixfold against *B. cereus* and *S. aureus* [50]).

Organic waste and heavy metal removal from wastewater have always been a major concern for the environment. In order to simultaneously remove trichlorfon and cadmium from an aqueous solution, Zhang et al., in 2020, created magnetic chitosan beads-immobilized *A. sydowii* [49]. The beads demonstrated considerable trichlorfon and cadmium removal capabilities, as well as outstanding four-cycle recyclability. As a result, the beads are appropriate and efficient for removing cadmium and trichlorfon simultaneously from wastewater [49].

## 6. Conclusions

Fungi have been subjected to much research due to their significance as wealth generators for various enzymes and bio-metabolites, as well as being intriguing for applications in agricultural, industrial, and pharmaceuticals fields.

**Figure 16:**
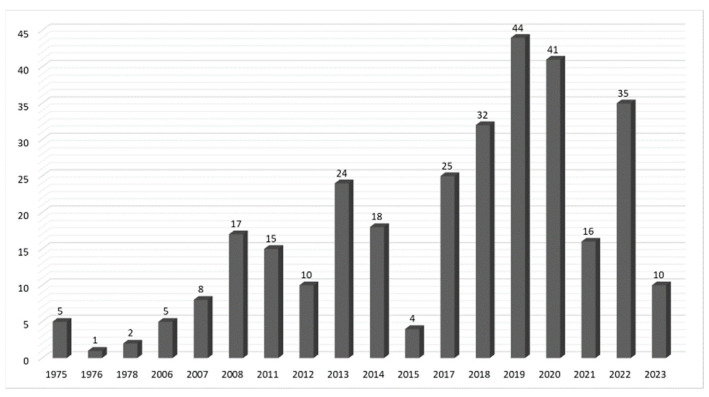
Number of metabolites reported from *A. sydowii* per year.

*A. sydowii* is a globally distributed fungus that was found to have the capacity to biosynthesize diverse classes of metabolites. In the current work, 246 metabolites were separated from *A. sydowii* in the period from 1975 to 2023 (Figure 16). Most of these metabolites were reported from 2017 to 2022.

**Figure 17:**
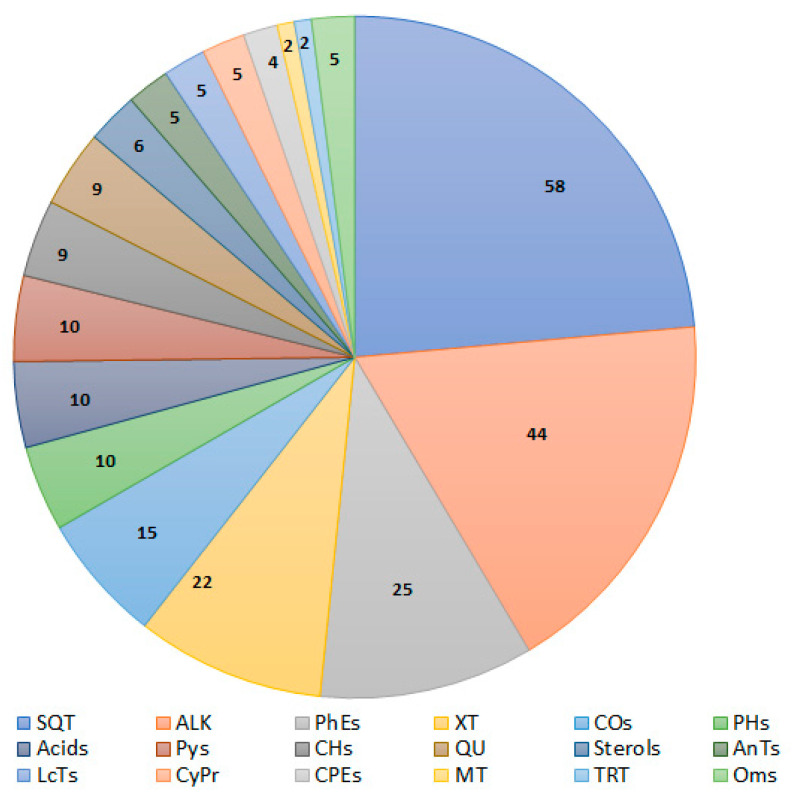
Different classes of metabolites reported from *A. sydowii.* AnTs: anthocyanins; SQT: sesquiterpenes; MT: monoterpenes; OMs: other metabolites; PHs: phenols; TRT: triterpens; ST: sterols: XT: xanthones; QU: quinones; ALK: alkaloids; PhEs: phenyl ethers; CHs: chromanes; COs: coumarins; Pys: pyranes; CPEs cyclopentenes; CyPr: cyclopropane, and lactone derivatives.

These metabolites include sesquiterpenoids, alkaloids, xanthones, monoterpenes, anthraquinones, sterols, triterpenes, phenyl ethers, pyrones, cyclopentenones, anthocyanins, coumarins, chromanes, acids, phenols, and other metabolites. Sesquiterpenoids (58 compounds, 24%), phenyl ethers (25 compounds, 10%), alkaloids (44 compounds, 18%), and xanthones (22 compounds, 9%) are the major constituents reported from this fungus (Figure 17).

This fungus was collected from different sources such as cultures, plants, marine environments (water, sea mud, sediment, gorgonian sea fans, algae, sponge, and driftwood), and liverworts. Most of the reported studies were carried out on *A. sydowii* isolated from marine sources. It is remarkable that this fungus has many enzymatic systems, which may help to explain why its metabolites are so diverse. Future studies will be useful in understanding the enzymes and genes responsible for the manufacture of these metabolites.

It was found that the coculture of this fungus with other microbes, as well as the modification of the culture media, significantly promoted the production of structurally varied metabolites, suggesting avenues of further research using these approaches for activating *A. sydowii*’s silent biosynthetic genes toward the accumulation of various substantial compounds.

**Figure 18:**
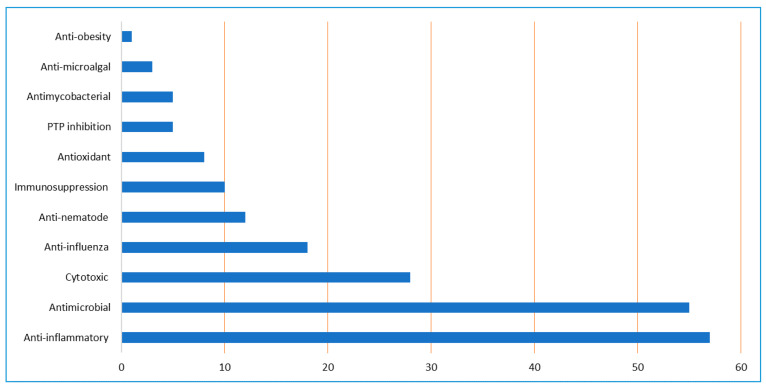
Number of metabolites evaluated for each bioactivity.

These metabolites were assessed for different bioactivities, including cytotoxic, antimicrobial, antioxidant, antiviral, anti-obesity, anti-inflammation, immunosuppression, anti-diabetic, protein tyrosine phosphatase 1B (PTP1B) inhibition, and anti-nematode activities (Figure 18).

Compounds **195** and **196** displayed potent antioxidant activity. Compounds **67**, **187**, **192**, and **239** demonstrated powerful cytotoxic potential. Compounds **2**, **3**, and **110** had notable antibacterial efficacy. Compounds **80**, **81**, **92**, **94**, and **234** displayed potent anti-influenza virus activity. Furthermore, compound **45** was found to possess anti-diabetic and anti-obesity capacities through promoting glucose consumption and suppressing lipid accumulation, whereas compound **178** had a potent PTP1B inhibition capacity compared to suramin, suggesting its possible application in anti-diabetic and anti-sleeping sickness therapeutic agents.

Despite the large number of metabolites, biological evaluation has only been conducted for a limited number of them, mainly in vitro, and there is a lack of pharmacological investigations that focus on studying the possible action mechanisms of the active metabolites. Therefore, mechanistic and in vivo studies are recommended to clarify and validate potential mechanisms for the active metabolites. Moreover, studies on the structure–activity relationships of these metabolites should be carried out.

Additionally, molecular dynamic and docking studies could be employed to investigate the possible bioactivities of the untested metabolites.

On the other side, many of the tested metabolites displayed no notable effectiveness in some of the tested activities. Therefore, estimation of other possible bioactivities and molecular dynamic and docking studies, as well as derivatization of these metabolites, should clearly be the target of future research.

For further production of structurally varied metabolites by this fungus, cocultivation techniques should be considered an area for future investigation. In addition, exploring the biosynthetic pathways of these bio-metabolites is required and could enable the rational engineering or refactoring of these pathways for industrial purposes. Further, identification of the biosynthetic genes responsible for these metabolites may provide the opportunity to discover *A. sydowii*’s genetic potential for discovering novel metabolites by metabolic engineering, which could lead to more affordable and novel pharmaceutics.

According to the published reports, *A. sydowii* can produce diverse types of enzymes with potential biotechnological and industrial applications. Research that focuses on engineering enzymes in such a way for maximum activity and stability under appropriate conditions is desirable. Recombinant DNA technology and engineering of proteins are required to improve the industrial production of these enzymes. *A. sydowii* can withstand high-salinity conditions, pointing to its biotechnological and industrial relevance. It was proven that this fungus adsorbed heavy metals and degraded pesticides, agrochemicals, and contaminants. As a result, *A. sydowii* might serve as an environmentally safe tool for bioremediation and for converting hazardous materials into useful products. The minor reports described NP synthesis utilizing this fungus. These biosynthesized NPs possessed antiproliferative and antimicrobial potential as well as biosorbent capacity for treating heavy metal- and pesticide-contaminated water. However, the synthesized NPs using *A. sydowii* are limited to silver, γ-Fe_2_O_3_ magnetic chitosan nanocomposites, and chitosan beads-immobilized *A. sydowii*. Therefore, future research should focus on developing protocols for implementing the biosynthesis of other types of NPs such as carbides, metal oxides, and nitrides using this fungus and their bio-evaluation, which could be a promising area for more anticipated beneficial effects.

Despite the large number of published studies on *A. sydowii*, mycologists, biologists, and chemists still need to conduct more extensive research to fully understand the potential of this fungus and its secondary metabolites.

## Figures and Tables

**Table 1:** Sesquiterpenoids reported from *Aspergillus sydowii* (molecular weight and formulae, strain, host, and location).

Compound Name	Mol. Wt.	Mol. Formula	Strain, Host, Location	Ref.
(+)-(7*S*)-Sydonic acid (**1**)	266	C_15_H_22_O_4_	Cultured, IFO 7531, Japan	[11]
	-	-	*Acanthophora spicifera* (red alga), Rameswaram, India	[53]
			Marine sediment, Hsinchu, Taiwan	[54]
	-	-	CUGB-F126, seawater, Bohai Sea, Tianjin	[15]
	-	-	C1-S01-A7, seawater sample, West Pacific Ocean	[55]
	-	-	PSU-F154, genus *Annella* sp. (gorgonian sea fan), coastal area, Surat Thani, Thailand	[56]
	-	-	MSX19583, spruce litter, Colorado, USA	[33]
	-	-	ZSDS1-F6, unidentified marine sponge, Xisha Islands, China	[45]
	-	-	C1-S01-A7, seawater sample, West Pacific Ocean	[55]
	-	-	SCSIO 41301, *Phakellia fusca* (marine sponge), Xisha Islands, China	[35]
	-	-	MCCC 3A00324, deep-sea sediment, South Atlantic Ocean	[57]
	-	-	Deep-sea mud, Dalian, China	[58]
	-	-	CPCC 401353, cultured, China	[59]
	-	-	LW09, deep-sea sediment, Southwest Indian Ridge	[47]
(7*S*)-(+)-Hydroxysydonic acid = Aspergoterpenin C (**2**)	282	C_15_H_22_O_5_	Cultured, IFO 7531, Japan	[11]
	-	-	*Acanthophora spicifera* (red alga), Rameswaram, India	[53]
	-	-	SP-1, marine sediment sample, Antarctic Great Wall Station	[40]
	-	-	EN-434, *Symphyocladia* *latiuscula* (red alga), Qingdao coastline, China	[32]
	-	-	MCCC 3A00324, deep-sea sediment, South Atlantic Ocean	[57]
	-	-	Piece of deep-sea mud, Dalian, China	[58]
	-	-	CPCC 401353, cultured, China	[59]
	-	-	LW09, deep-sea sediment, Southwest Indian Ridge	[47]
(7*S*)-(−)-10-Hydroxysydonic acid (**3**)	282	C_15_H_22_O_5_	Piece of deep-sea mud, Dalian, China	[58]
	-	-	MCCC 3A00324, deep-sea sediment, South Atlantic Ocean	[57]
	-	-	CPCC 401353, cultured, China	[59]
(+)-(7*S*)-7-O-Methylsydonic acid (**4**)	280	C_16_H_24_O_4_	PSU-F154, genus *Annella* sp. (marine gorgonian sea fan), coastal area, Surat Thani, Thailand	[56]
(7*S*,11*S*)-(+)-12-Hydroxysydonic acid (**5**)	282	C_15_H_22_O_5_	Marine sediment, Hsinchu, Taiwan	[54]
	-	-	SP-1, marine sediment, Antarctic Great Wall Station	[40]
	-	-	SCSIO 41301, *Phakellia fusca* (marine sponge), Xisha Islands, China	[35]
	-	-	LW09, deep-sea sediment, Southwest Indian Ridge	[47]
(7*S*,11*S*)-(+)-12-Acetoxysydonic acid (**6**)	324	C_17_H_24_O_6_	ZSDS1-F6, unidentified marine sponge, Xisha Islands, China	[45]
(S)-(+)-Dehydrosydonic acid (**7**)	264	C_15_H_20_O_4_	ZSDS1-F6, unidentified marine sponge, Xisha Islands, China	[45]
7-Deoxy-7,14-didehydrosydonic acid (**8**)	248	C_15_H_20_O_3_	CUGB-F126, seawater, Bohai Sea, Tianjin	[15]
	-	-	SCSIO 41301, *Phakellia fusca* (marine sponge), Xisha Islands, China	[35]
(E)-7-deoxy-7,8-didehydrosydonic acid (**9**)	248	C_15_H_20_O_3_	SCSIO 41301, *Phakellia fusca* (marine sponge), Xisha Islands, China	[35]
(Z)-7-deoxy-7,8-didehydrosydonic acid (**10**)	248	C_15_H_20_O_3_	SCSIO 41301, marine sponge *Phakellia fusca*, Xisha Islands, China	[35]
(−)-(R)-Cyclohydroxysydonic acid (**11**)	280	C_15_H_20_O_5_	LW09, deep-sea sediment, Southwest Indian Ridge	[47]
Penicibisabolane G (**12**)	264	C_15_H_20_O_4_	LW09, deep-sea sediment, Southwest Indian Ridge	[47]
11,12-Dihydroxysydonic acid (**13**)	298	C_15_H_22_O_6_	LW09, deep-sea sediment, Southwest Indian Ridge	[47]
Expansol G (**14**)	324	C_17_H_24_O_6_	LW09, deep-sea sediment, Southwest Indian Ridge	[47]
Aspergillusene C (**15**)	264	C_15_H_20_O_4_	ZSDS1-F6, unidentified marine sponge, Xisha Islands, China	[45]
Aspergillusene D (**16**)	250	C_15_H_22_O_3_	SCSIO 41301, *Phakellia fusca* (marine sponge), Xisha Islands, China	[35]
Methyl (S)-(3-Hydroxy-4-(2-hydroxy-6-methylheptan-2-yl)benzoyl)glycinate = (+)-(7*S*)-Sydonic acid glycinate (**17**)	337	C_18_H_27_NO_5_	CUGB-F126, seawater, Bohai Sea, Tianjin	[15]
Serine sydonate (**18**)	353	C_18_H_27_NO_6_	Deep-sea mud, Dalian, China	[58]
	-	-	Cultured, CPCC 401353, China	[59]
4′-Alkenyl serine sydonate (**19**)	351	C_18_H_25_NO_6_	Deep-sea mud, Dalian, China	[58]
4′-Hydroxyl serine sydonate (**20**)	369	C_18_H_27_NO_7_	Deep-sea mud, Dalian, China	[58]
5′-Hydroxyl serine sydonate (**21**)	369	C_18_H_27_NO_7_	Deep-sea mud, Dalian, China	[58]
cyclo-12-Hydroxysydonic acid (**22**)	264	C_15_H_20_O_4_	SCSIO 41301, *Phakellia fusca* (marine sponge), Xisha Islands, China	[35]
Sydowic acid (**23**)	264	C_15_H_20_O_4_	Cultured, Japan	[27,29,30]
	-	-	IFO 4284, cultured, Japan	[29,30]
	-	-	*Acanthophora spicifera* (red alga), Rameswaram, India	[53]
	-	-	CUGB-F126, seawater, Bohai Sea, Tianjin	[15]
	-	-	C1-S01-A7, seawater sample, West Pacific Ocean	[55]
	-	-	EN-434, *Symphyocladia* *latiuscula* (red alga), Qingdao coastline, China	[32]
	-	-	*Rhododendron mole* (leaves), Xing’an, Guangxi, China	[26]
(7*S*,8*S*)-8-Hydroxysydowic acid (**24**)	280	C_15_H_20_O_5_	EN-434, *Symphyocladia* *latiuscula* (red alga), Qingdao coastline, China	[32]
(±)-(7R*,10R*)-10-Hydroxysydowic acid (**25**)	280	C_15_H_20_O_5_	EN-434, *Symphyocladia* *latiuscula* (red alga), Qingdao coastline, China	[32]
(−)-(7*R*,10*S*)-10-Hydroxysydowic acid (**26**)	280	C_15_H_20_O_5_	MCCC 3A00324, deep-sea sediment, South Atlantic Ocean	[57]
	-	-	*Rhododendron mole* (leaves), Xing’an, Guangxi, China	[26]
(−)-(7*R*,10*R*)-iso-10-Hydroxysydowic acid (**27**)	280	C_15_H_20_O_5_	MCCC 3A00324, deep-sea sediment, South Atlantic Ocean	[57]
Asperbisabolane A (**28**)	278	C_15_H_18_O_5_	MCCC 3A00324, deep-sea sediment, South Atlantic Ocean	[57]
Asperbisabolane B (**29**)	292	C_15_H_16_O_6_	MCCC 3A00324, deep-sea sediment, South Atlantic Ocean	[57]
Asperbisabolane C (**30**)	280	C_15_H_20_O_5_	MCCC 3A00324, deep-sea sediment, South Atlantic Ocean	[57]
Asperbisabolane D (**31**)	278	C_15_H_18_O_5_	MCCC 3A00324, deep-sea sediment, South Atlantic Ocean	[57]
Asperbisabolane E (**32**)	280	C_15_H_20_O_5_	MCCC 3A00324, deep-sea sediment, South Atlantic Ocean	[57]
Asperbisabolane F (**33**)	278	C_15_H_18_O_5_	MCCC 3A00324, deep-sea sediment, South Atlantic Ocean	[57]
Asperbisabolane G (**34**)	280	C_15_H_20_O_5_	MCCC 3A00324, deep-sea sediment, South Atlantic Ocean	[57]
Asperbisabolane H (**35**)	280	C_15_H_20_O_5_	MCCC 3A00324, deep-sea sediment, South Atlantic Ocean	[57]
Asperbisabolane I (**36**)	280	C_15_H_20_O_5_	MCCC 3A00324, deep-sea sediment, South Atlantic Ocean	[57]
Asperbisabolane J (**37**)	264	C_14_H_16_O_5_	MCCC 3A00324, deep-sea sediment, South Atlantic Ocean	[57]
Asperbisabolane K (**38**)	284	C_13_H_16_O_5_S	MCCC 3A00324, deep-sea sediment, South Atlantic Ocean	[57]
Asperbisabolane L (**39**)	206	C_12_H_14_O_3_	MCCC 3A00324, deep-sea sediment, South Atlantic Ocean	[57]
Asperbisabolane M (**40**)	280	C_15_H_20_O_5_	MCCC 3A00324, deep-sea sediment, South Atlantic Ocean	[57]
Asperbisabolane N (**41**)	340	C_17_H_24_O_7_	MCCC 3A00324, deep-sea sediment, South Atlantic Ocean	[57]
Aspergillusene A = (E)-5-(Hydroxymethyl)-2-(6′-methylhept-2′-en-2′-yl)phenol (**42**)	234	C_15_H_22_O_2_	PSU-F154, marine gorgonian sea fan of the genus *Annella* sp., coastal area, Surat Thani, Thailand	[56]
	-	-	Marine sediment, Hsinchu, Taiwan	[54]
	-	-	ZSDS1-F6, unidentified marine sponge, Xisha Islands, China	[45]
	-	-	MCCC 3A00324, deep-sea sediment, South Atlantic Ocean	[57]
	-	-	LW09, deep-sea sediment, Southwest Indian Ridge	[47]
Aspergillusene B (**43**)	246	C_15_H_18_O_3_	PSU-F154, genus *Annella* sp. (gorgonian sea fan), coastal area, Surat Thani, Thailand	[56]
	-	-	LW09, deep-sea sediment, Southwest Indian Ridge	[47]
β-D-Glucopyranosyl aspergillusene A (**44**)	396	C_21_H_32_O_7_	J05B-7F-4, *Stelletta* sp. (marine sponge), South Korea	[36]
(+)-(7*S*)-Sydonol (**45**)	252	C_15_H_24_O_3_	MSX19583, spruce litter, Colorado, USA	[33]
	-	-	Marine sediment, Hsinchu, Taiwan	[54]
	-	-	MCCC 3A00324, deep-sea sediment, South Atlantic Ocean	[57]
(+)-(7*S*)-7-O-Methylsydonol (**46**)	266	C_16_H_26_O_3_	Marine sediment, Hsinchu, Taiwan	[54]
7-Deoxy-7,14-didehydrosydonol (**47**)	234	C_15_H_22_O_2_	Marine sediment, Hsinchu, Taiwan	[54]
	-	-	MCCC 3A00324, deep-sea sediment, South Atlantic Ocean	[57]
(−)-5-(hydroxymethyl)-2-(2′,6′,6′-trimethyltetrahydro-2H-pyran-2-yl)phenol (**48**)	250	C_15_H_22_O_3_	*Rhododendron mole* (leaves), Xing’an, Guangxi, China	[26]
Anhydrowaraterpol B (**49**)	250	C_15_H_22_O_3_	Marine sediment, Hsinchu, Taiwan	[54]
	-	-	ZSDS1-F6, unidentified marine sponge, Xisha Islands, China	[45]
(Z)-5-(Hydroxymenthyl)-2-(6′)-methylhept-2′-en-2′-yl)-phenol (**50**)	234	C_15_H_22_O_2_	ZSDS1-F6, unidentified marine sponge, Xisha Islands, China	[45]
	-	-	MCCC 3A00324, deep-sea sediment, South Atlantic Ocean	[57]
Methyl(R,E)-6-(2,3-dihydroxy-4-methylpenyl)-2-methylhept-5-enoate (**51**)	278	C_16_H_22_O_4_	SW9, seawater sample, Yangma Island, Yantai, China	[41]
Cyclowaraterpol A (**52**)	250	C_15_H_22_O_3_	ZSDS1-F6, unidentified marine sponge, Xisha Islands, China	[45]
(7*S*)-Flavilane A (**53**)	298	C_16_H_26_O_3_S	10–31, deep-sea sediments, cold seep off southwestern Taiwan	[38]
(7*S*)-4-Iodo-flavilane A (**54**)	424	C_16_H_25_IO_3_S	10–31, deep-sea sediments, cold seep off southwestern Taiwan	[38]
Aspersydosulfoxide A (**55**)	280	C_16_H_24_O_2_S	LW09, deep-sea sediment, Southwest Indian Ridge	[47]
Aspercuparene A (**56**)	262	C_15_H_18_O_4_	MCCC 3A00324, deep-sea sediment, South Atlantic Ocean	[57]
Aspercuparene B (**57**)	264	C_15_H_20_O_4_	MCCC 3A00324, deep-sea sediment, South Atlantic Ocean	[57]
Aspercuparene C (**58**)	260	C_15_H_16_O_4_	MCCC 3A00324, deep-sea sediment, South Atlantic Ocean	[57]

**Table 2:** Mono- and triterpenoids and sterols reported from *A. sydowii* (molecular weight and formulae, strain, host, and location).

Compound Name	Mol. Wt.	Mol. Formula	Strain, Host, Location	Ref.
Monoterpenoids				
Aspermonoterpenoid A (**59**)	198	C_10_H_14_O_4_	MCCC 3A00324, deep-sea sediment, South Atlantic Ocean	[60]
Aspermonoterpenoid B (**60**)	182	C_10_H_14_O_3_	MCCC 3A00324, deep-sea sediment, South Atlantic Ocean	[60]
Triterpenoids				
(4*S*,5*S*,6*S*,8*S*,9*S*,10*R*,13*R*,14*S*,16*S*,17*Z*)-6,16-Diacetoxy-25-hydroxy-3,7-dioxy-29-nordammara-1,17(20)-dien-21-oic acid (**61**)	572	C_32_H_44_O_9_	PFW1-13, driftwood, beach of Baishamen, Hainan, China	[48]
Helvolic acid (**62**)	554	C_32_H_42_O_8_	PFW1-13, driftwood, beach of Baishamen, Hainan, China	[48]
Sterols				
Ergosterol peroxide (**63**)	430	C_28_H_46_O_3_	C1-S01-A7, seawater, West Pacific Ocean	[55]
Ergosta-7,22-dien-3β-ol (**64**)	398	C_28_H_46_O	C1-S01-A7, seawater, West Pacific Ocean	[55]
Ergosterol (**65**)	396	C_28_H_44_O	C1-S01-A7, seawater, West Pacific Ocean	[55]
β-Sitosterol (**66**)	414	C_29_H_50_O	C1-S01-A7, seawater, West Pacific Ocean	[55]
Cerevisterol (**67**)	430	C_28_H_46_O_3_	YH11-2, deep-sea fungus, Guam, South Japan	[44]
(17*R*)-17-Methylincistererol (**68**)	346	C_22_H_34_O_3_	YH11-2, deep-sea fungus, Guam, South Japan	[44]

**Table 3:** Xanthones and quinones reported from *Aspergillus sydowii* (molecular weight and formulae, strain, host, and location).

Compound Name/Chemical Class	Mol. Wt.	Mol. Formula	Strain, Host, and Location	Ref.
Xanthones				
Sydowinin A (**69**)	300	C_16_H_12_O_6_	Cultured, IFO 4284, Japan	[29]
	-	-	PSU-F154, genus *Annella* sp. (gorgonian sea fan), coastal area, Surat Thani, Thailand	[56]
12-O-Acetyl-sydowinin A (**70**)	342	C_18_H_14_O_7_	C1-S01-A7, seawater, West Pacific Ocean	[55]
Sydowinin B (**71**)	316	C_16_H_12_O_7_	Cultured, IFO 4284, Japan	[29]
	-	-	Marine sediment, Hsinchu, Taiwan	[54]
	-	-	PSU-F154, genus *Annella* sp. (gorgonian sea fan), coastal area, Surat Thani, Thailand	[56]
	-	-	Marine sediment, Hsinchu, Taiwan	[54]
	-	-	C1-S01-A7, seawater, West Pacific Ocean	[55]
13-O-Acetylsydowinin B (**72**)	358	C_18_H_14_O_8_	*Scapania ciliata* (Chinese liverwort), Maoer Mountain, Guangxi, China	[63]
	-	-	J05B-7F-4, *Stelletta* sp. (marine sponge), South Korea	[36]
	-	-	C1-S01-A7, seawater, West Pacific Ocean	[55]
Methyl 8-hydroxy-6-methyl-9-oxo-9*H*-xanthene-1-carboxylate (**73**)	284	C_16_H_12_O_5_	PSU-F154, genus *Annella* sp. (gorgonian sea fan), coastal area, Surat Thani, Thailand	[56]
Pinselin (**74**)	300	C_16_H_12_O_6_	PSU-F154, genus *Annella* sp. (gorgonian sea fan), coastal area, Surat Thani, Thailand	[56]
	-	-	*Scapania ciliata* (Chinese liverwort), Maoer Mountain, Guangxi, China	[63]
	-	-	C1-S01-A7, seawater, West Pacific Ocean	[55]
Methyl 1,6-dihydroxy-3-methyl-9-oxo-9*H*-xanthene-1-carboxylate (**75**)	300	C_16_H_12_O_6_	*Scapania ciliata* (Chinese liverwort), Maoer Mountain, Guangxi, China	[56]
Sydoxanthone A (**76**)	388	C_19_H_16_O_7_S	*Scapania ciliata* (Chinese liverwort), Maoer Mountain, Guangxi, China	[63]
Sydoxanthone B (**77**)	346	C_17_H_14_O_6_S	*Scapania ciliata* (Chinese liverwort), Maoer Mountain, Guangxi, China	[63]
8-Hydroxy-6-methyl-9-oxo-9*H*-xanthene-1-carboxylic acid methyl ester (**78**)	284	C_16_H_12_O_5_	*Scapania ciliata* (Chinese liverwort), Maoer Mountain, Guangxi, China	[63]
2-Hydroxy-6-formyl-vertixanthone (**79**)	314	C_16_H_10_O_7_	C1-S01-A7, seawater, West Pacific Ocean	[55]
2-Hydroxy-1-(hydroxymethyl)-8-methoxy-3-methyl-9*H*-xanthen-9-one (**80**)	286	C_16_H_14_O_5_	SCSIO 41301, *Phakellia fusca* (marine sponge), Xisha Islands, China	[35]
2-Hydroxy-1-(hydroxymethyl)-7,8-dimethoxy-3-methyl-9*H*-xanthen-9-one (**81**)	316	C_17_H_16_O_6_	SCSIO 41301, *Phakellia fusca* (marine sponge), Xisha Islands, China	[35]
Austocystin A (**82**)	372	C_19_H_13_ClO_6_	SCSIO 00305, *Verrucella unbracculum* (gorgonian), South China Sea, Sanya, Hainan, China	[24]
6-Methoxyl austocystin A (**83**)	402	C_20_H_15_ClO_7_	SCSIO 00305, *Verrucella unbracculum* (gorgonian), South China Sea, Sanya, Hainan, China	[24]
Sterigmatocystin (**84**)	324	C_18_H_12_O_6_	DC08, *Dachtylospongia* sp. (marine sponge), South Coast, West Sumatra, Indonesia	[39]
Sydowinol (**85**)	318	C_16_H_14_O_7_	IFO 4284, Cultured, Japan	[29]
Aspergillusone A (**86**)	304	C_16_H_16_O_6_	PSU-F154, genus *Annella* sp. (gorgonian sea fan), coastal area, Surat Thani, Thailand	[56]
	-	-	C1-S01-A7, seawater, West Pacific Ocean	[55]
Aspergillusone B (**87**)	338	C_16_H_18_O_8_	PSU-F154, genus *Annella* sp. (gorgonian sea fan), coastal area, Surat Thani, Thailand	[56]
(7*R*,8*R*)-AGI-B4 (**88**)	320	C_16_H_16_O_7_	PSU-F154, genus *Annella* sp. (gorgonian sea fan), coastal area, Surat Thani, Thailand	[56]
	-	-	Marine sediment, Hsinchu, Taiwan	[54]
	-	-	C1-S01-A7, seawater, West Pacific Ocean	[55]
12-O-Acetyl (7*R*,8*R*)-AGI-B4 (**89**)	362	C_18_H_18_O_8_	C1-S01-A7, seawater, West Pacific Ocean	[55]
(7*R*,8*R*)-α-Diversonolic ester (**90**)	322	C_16_H_18_O_7_	PSU-F154, genus *Annella* sp. (gorgonian sea fan), coastal area, Surat Thani, Thailand	[56]
Quinones				
Emodin (**91**)	270	C_15_H_10_O_5_	*Scapania ciliata* (Chinese liverwort), Maoer Mountain, Guangxi, China	[63]
	-	-	C1-S01-A7, seawater, West Pacific Ocean	[55]
Emodic acid (**92**)	300	C_15_H_8_O_7_	SCSIO 41301, *Phakellia fusca* (marine sponge), Xisha Islands, China	[35]
Parietinic acid (**93**)	314	C_16_H_10_O_7_	SCSIO 41301, *Phakellia fusca* (marine sponge), Xisha Islands, China	[35]
Questin (**94**)	284	C_16_H_12_O_5_	*Scapania ciliata* (Chinese liverwort), Maoer Mountain, Guangxi, China	[63]
	-	-	C1-S01-A7, seawater, West Pacific Ocean	[55]
	-	-	SCSIO 41301, marine sponge *Phakellia fusca*, Xisha Islands, China	[35]
1,6,8-Trihydroxy-3-methylanthraquinone (**95**)	270	C_15_H_10_O_5_	SCSIO 41301, marine sponge *Phakellia fusca*, Xisha Islands, China	[35]
Yicathin C (**96**)	312	C_17_H_12_O_6_	C1-S01-A7, seawater sample, West Pacific Ocean	[55]
1-Hydroxy-6,8-dimethoxy-3-methylanthraquinone (**97**)	298	C_17_H_14_O_5_	*Scapania ciliata* (Chinese liverwort), Maoer Mountain, Guangxi, China	[63]
(+)-3,3′,7,7′,8,8′-hexahydroxy-5,5′-dimethyl-bianthra-quinone (**98**)	538	C_30_H_18_O_10_	#2B, leaves, *Aricennia marina*, Yangjiang, Guangdong, China	[64]
Xanthoradone A (**99**)	490	C_27_H_22_O_9_	#2B, leaves, *Aricennia marina*, Yangjiang, Guangdong, China	[64]

**Table 4:** Alkaloids reported from *Aspergillus sydowii* (molecular weight and formulae, strain, host, and location).

Compound Name	Mol. Wt.	Mol. Formula	Strain, Host, and Location	Ref.
Cyclotryprostatin B (**100**)	425	C_23_H_27_N_3_O_5_	SCSIO 00305, *Verrucella umbraculum* (gorgonian), Sanya, Hainan, China	[31]
Cyclotryprostatin E (**101**)	443	C_23_H_29_N_3_O_6_	SCSIO 00305, *Verrucella umbraculum* (gorgonian), Sanya, Hainan, China	[31]
Fumitremorgin B (**102**)	479	C_27_H_33_N_3_O_5_	SCSIO 00305, *Verrucella umbraculum* (gorgonian), Sanya, Hainan, China	[31]
6-Methoxyspirotryprostatin B (**103**)	393	C_22_H_23_N_3_O_4_	PFW1-13, driftwood, Baishamen beach, Hainan, China	[48]
18-Oxotryprostatin A (**104**)	395	C_22_H_25_N_3_O_4_	PFW1-13, driftwood, Baishamen beach, Hainan, China	[48]
14-Hydroxyterezine D (**105**)	341	C_19_H_23_N_3_O_3_	PFW1-13, driftwood, Baishamen beach, Hainan, China	[48]
Spirotryprostatin A (**106**)	365	C_21_H_23_N_3_O_2_	PFW1-13, driftwood, Baishamen beach, Hainan, China	[48]
Terezine D (**107**)	325	C_19_H_23_N_3_O_2_	PFW1-13, driftwood, Baishamen beach, Hainan, China	[48]
Fumitremorgin C (**108**)	379	C_22_H_25_N_3_O_3_	PFW1-13, driftwood, Baishamen beach, Hainan, China	[48]
12,13-Dihydroxyfumitremorgin C (**109**)	411	C_22_H_25_N_3_O_5_	PFW1-13, driftwood, Baishamen beach, Hainan, China	[48]
(11*S*,14*S*)-Cyclo-(L-Trp-L-Phe) (**110**)	333	C_20_H_19_N_3_O_2_	PSU-F154, genus *Annella* sp. (gorgonian sea fan), coastal area, Surat Thani, Thailand	[56]
	-	-	MSX19583, spruce litter, Colorado, USA	[33]
	-	-	J05B-7F-4, *Stelletta* sp. (marine sponge), South Korea	[36]
	-	-	ZSDS1-F6, unidentified marine sponge, Xisha Islands, China	[45]
	-	-	SP-1, marine sediment, Antarctic Great Wall Station	[40]
	-	-	MCCC 3A00324, deep-sea sediment, South Atlantic Ocean	[65]
Didehydrobisdethiobis(methylthio)gliotoxin (**111**)	356	C_15_H_20_N_2_O_4_S_2_	PFW1-13, driftwood, Baishamen beach, Hainan, China	[48]
Verruculogen (**112**)	354	C_15_H_18_N_2_O_4_S_2_	PFW1-13, driftwood, Baishamen beach, Hainan, China	[48]
Cyclo-(S-Pro-S-Ile) (**114**)	210	C_11_H_18_N_2_O_2_	Cultured, China	[28]
Cyclo-(S-Pro-R-Leu) (**113**)	210	C_11_H_18_N_2_O_2_	Cultured, China	[28]
WIN 64821 (**115**)	664	C_40_H_36_N_6_O_4_	MSX19583, spruce litter, Colorado, USA	[33]
	-	-	C1-S01-A7, seawater, West Pacific Ocean	[55]
Bisdethiobis(methylthio)-acetylaranotin (**116**)	534	C_24_H_26_N_2_O_8_S_2_	Cultured, China	[28]
[4-(2-Methoxyphenyl)-1-piperazinyl][(1-methyl-1*H*-indol-3-yl)]-methanone (**117**)	349	C_21_H_23_N_3_O_2_	SCSIO 00305, *Verrucella umbraculum* (gorgonian), Sanya, Hainan, China	[31]
Fumiquinazoline A (**118**)	445	C_24_H_23_N_5_O_4_	SCSIO 00305, *Verrucella umbraculum* (gorgonian), Sanya, Hainan, China	[31]
Fumiquinazoline B (**119**)	445	C_24_H_23_N_5_O_4_	SCSIO 00305, *Verrucella umbraculum* (gorgonian), Sanya, Hainan, China	[31]
Fumiquinazoline C (**120**)	443	C_24_H_21_N_5_O_4_	SCSIO 00305, *Verrucella umbraculum* (gorgonian), Sanya, Hainan, China	[31]
Fumiquinazoline D (**121**)	443	C_24_H_21_N_5_O_4_	SCSIO 00305, *Verrucella umbraculum* (gorgonian), Sanya, Hainan, China	[31]
Fumiquinazoline F (**122**)	358	C_21_H_18_N_4_O_2_	SCSIO 00305, *Verrucella umbraculum* (gorgonian), Sanya, Hainan, China	[31]
Fumiquinazoline G (**123**)	358	C_21_H_18_N_4_O_2_	SCSIO 00305, *Verrucella umbraculum* (gorgonian), Sanya, Hainan, China	[31]
2-(4-Hydroxybenzyl)-4-(3-acetyl)quinazolin-one (**124**)	294	C_17_H_14_N_2_O_3_	SW9, seawater, Yangma Island, Yantai, China	[41]
2-(4-Hydroxybenzoyl)-4(3*H*)-quinazolinone (**125**)	252	C_15_H_12_N_2_O_2_	SW9, seawater, Yangma Island, Yantai, China	[41]
2-(4-Oxo-3,4-dihydroquinazolin-2-yl)benzoic acid (**126**)	266	C_15_H_10_N_2_O_3_	MCCC 3A00324, deep-sea sediment, South Atlantic Ocean	[65]
Acremolin (**127**)	231	C_11_H_13_N_5_O	MCCC 3A00324, deep-sea sediment, South Atlantic Ocean	[65]
Acremolin C (**128**)	245	C_12_H_15_N_5_O	SP-1, marine sediment, Antarctic Great Wall Station	[40]
Acremolin D (**129**)	289	C_13_H_15_N_5_O_3_	MCCC 3A00324, deep-sea sediment, South Atlantic Ocean	[65]
Pseustin A (**130**)	431	C_22_H_25_NO_8_	PFW1-13, driftwood, Baishamen beach, Hainan, China	[48]
14-Norpseurotin A (**131**)	417	C_21_H_23_NO_8_	PFW1-13, driftwood, Baishamen beach, Hainan, China	[48]
Azaspirofurans A (**132**)	411	C_21_H_19_NO_7_	D2-6, Marine sediment, Jiaozhou Bay, China	[43]
Azaspirofurans B (**133**)		C_22_H_21_NO_7_	D2-6, Marine sediment, Jiaozhou Bay, China	[43]
Chrysotriazole A (**134**)	311	C_17_H_17_N_3_O_3_	SW9, seawater, Yangma Island, Yantai, China	[41]
Indoleacetic acid (**135**)	175	C_10_H_9_NO_2_	MCCC 3A00324, deep-sea sediment, South Atlantic Ocean	[65]
Pyrrole-2-carboxylic acid (**136**)	111	C_5_H_5_NO_2_	MCCC 3A00324, deep-sea sediment, South Atlantic Ocean	[65]
2-Acetylaminobenzamide (**137**)	178	C_9_H_10_N_2_O_2_	C1-S01-A7, seawater, West Pacific Ocean	[55]
1,4-Dioxa-9,12-diazacyclohexadecane-5,8,13,16-tetraone (**138**)	286	C_12_H_18_N_2_O_6_	Cultured, China	[28]
N-Acetyltyramine (**139**)	179	C_10_H_13_NO_2_	Cultured, China	[28]
Fumigaclavine B (**140**)	366	C_23_H_30_N_2_O_2_	PFW1-13, driftwood, Baishamen beach, Hainan, China	[48]
Fumigaclavine C (**141**)	298	C_18_H_22_N_2_O_2_	PFW1-13, driftwood, Baishamen beach, Hainan, China	[48]
Pyripyropene A (**142**)	525	C_29_H_35_NO_8_	PFW1-13, driftwood, Baishamen beach, Hainan, China	[48]
Pyripyropene E (**143**)	569	C_30_H_35_NO_10_	PFW1-13, driftwood, Baishamen beach, Hainan, China	[48]

**Table 5:** Phenyl ether derivatives reported from *Aspergillus sydowii* (molecular weight and formulae, strain, host, and location).

Compound Name	Mol. Wt.	Mol. Formula	Strain, Host, Location	Ref.
Violaceol I (**144**)	262	C_14_H_14_O_5_	MF357, sea sediment, East China Sea, China	[37]
	-	-	J05B-7F-4, *Stelletta* sp. (marine sponge), South Korea	[36]
Violaceol II (**145**)	248	C_13_H_12_O_5_	MF357, sea sediment, East China Sea, China	[37]
	-	-	J05B-7F-4, *Stelletta* sp. (marine sponge), South Korea	[36]
Diorcinol (**146**)	230	C_14_H_14_O_3_	Marine sediment, Hsinchu, Taiwan	[54]
	-	-	J05B-7F-4, *Stelletta* sp. (marine sponge), South Korea	[36]
	-	-	FNA026, seawater, Xiamen, China	[9]
	-	-	MCCC 3A00324, deep-sea sediment, South Atlantic Ocean	[60]
4-Carboxydiorcinal (**147**)	274	C_15_H_14_O_5_	J05B-7F-4, *Stelletta* sp. (marine sponge), South Korea	[36]
			FNA026, seawater, Xiamen, China	[9]
Diorcinolic acid (**148**)	318	C_16_H_14_O_7_	J05B-7F-4, *Stelletta* sp. (marine sponge), South Korea	[36]
Glyceryl diorcinolic acid (**149**)	392	C_19_H_20_O_9_	FNA026, seawater, Xiamen, China	[9]
4-Methoxycarbonyl diorcinol (**150**)	288	C_16_H_16_O_5_	FNA026, seawater, Xiamen, China	[9]
10-Deoxygerfelin (**151**)	274	C_15_H_14_O_5_	CPCC 401353, cultured, China	[59]
Cordyol C (**152**)	246	C_14_H_14_O_4_	J05B-7F-4, *Stelletta* sp. (marine sponge), South Korea	[36]
	-	-	FNA026, seawater, Xiamen, China	[9]
	-	-	MCCC 3A00324, deep-sea sediment, South Atlantic Ocean	[60]
Cordyol E (**153**)	244	C_15_H_16_O_3_	J05B-7F-4, *Stelletta* sp. (marine sponge), South Korea	[36]
Cordyol F (**154**)	276	C_15_H_16_O_5_	FNA026, seawater, Xiamen, China	[9]
Cordyol C-3-O-α-D-ribofuranoside (**155**)	378	C_19_H_22_O_8_	FNA026, seawater, Xiamen, China	[9]
Diorcinol-3-O-*α*-D-ribofuranoside (**156**)	362	C_19_H_22_O_7_	FNA026, seawater, Xiamen, China	[9]
4-Methoxycarbonyl diorcinol-3-O-α-D-glucoside (**157**)	450	C_22_H_26_O_10_	FNA026, seawater, Xiamen, China	[9]
Disydonol B (**158**)	486	C_30_H_46_O_5_	FNA026, seawater, Xiamen, China	[55]
2-(Ethoxycarbonyl)-4′-carboxydiorcinal (**159**)	348	C_17_H_16_O_8_	FNA026, seawater, Xiamen, China	[9]
7-Ethyldiorcinol (**160**)	244	C_15_H_16_O_3_	FNA026, seawater, Xiamen, China	[9]
3-Hydroxydiorcinol (**161**)	246	C_14_H_14_O_4_	FNA026, seawater, Xiamen, China	[9]
Aspergilol E (**162**)	304	C_16_H_16_O_6_	FNA026, seawater, Xiamen, China	[9]
4-Hydroxy-2-(3′-hydroxy-4-methoxycarbonyl-5′-methylphenoxy)-6-methylbenzoic acid (**163**)	332	C_17_H_16_O_7_	FNA026, seawater, Xiamen, China	[9]
Aspermutarubrol (**164**)	262	C_14_H_14_O_5_	FNA026, seawater, Xiamen, China	[9]
Bisviolaceol II (**165**)	506	C_28_H_26_O_9_	10–31, sediments, deep-sea, cold seep off southwestern Taiwan	[38]
Sydowiol A (**166**)	370	C_20_H_18_O_7_	MF357, sea sediment, East China Sea, China	[37]
Sydowiol B (**167**)	384	C_21_H_20_O_7_	MF357, sea sediment, East China Sea, China	[37]
Sydowiol C (**168**)	384	C_21_H_20_O_7_	MF357, sea sediment, East China Sea, China	[37]

**Table 6:** Chromane and coumarin derivatives reported from *Aspergillus sydowii* (molecular weight and formulae, strain, host, and location).

Compound Name	Mol. Wt.	Mol. Formula	Strain, Host, Location	Ref.
Citrinin (**169**)	250	C_13_H_14_O_5_	EN-534, *Laurencia okamurai* (red alga), Qingdao, China	[69]
Penicitrinol A (**170**)	382	C_23_H_26_O_5_	EN-534, *Laurencia okamurai* (red alga), Qingdao, China	[[6]
*seco*-Penicitrinol A (**171**)	398	C_23_H_26_O_6_	EN-534, *Laurencia okamurai* (red alga), Qingdao, China	[69]
Penicitrinol L (**172**)	266	C_14_H_18_O_5_	EN-534, *Laurencia okamurai* (red alga), Qingdao, China	[69]
Penicitrinone A (**173**)	380	C_23_H_24_O_5_	EN-534, *Laurencia okamurai* (red alga), Qingdao, China	[69]
Penicitrinone F (**174**)	394	C_24_H_26_O_5_	EN-534, *Laurencia okamurai* (red alga), Qingdao, China	[69]
Dihydrocitrinone (**175**)	266	C_13_H_14_O_6_	EN-534, *Laurencia okamurai* (red alga), Qingdao, China	[69]
Decarboxydihydrocitrinone (**176**)	222	C_12_H_14_O_4_	EN-534, *Laurencia okamurai* (red alga), Qingdao, China	[69]
(−)-Asperentin (**177**)	292	C_16_H_20_O_5_	F00785, *Enteromorpha prolifera* (green alga), Jinjiang Saltern, Fujian province, China	[70]
			LF660, sea sediment, Mediterranean Sea, Levantine Basin SE of Crete	[46]
Asperentin B (**178**)	308	C_16_H_20_O_6_	LF660, sea sediment, Mediterranean Sea, Levantine Basin SE of Crete	[46]
5-*O*-Methyl-asperentin B = 5-Hydroxyl-6-*O*-methylasperentin (**179**)	322	C_17_H_22_O_6_	F00785, *Enteromorpha prolifera* (green alga), Jinjiang Saltern, Fujian province, China	[70]
			LF660, sea sediment, Mediterranean Sea, Levantine Basin SE of Crete	[46]
6-*O*-α-D-Ribosylasperentin (**180**)	424	C_21_H_28_O_9_	F00785, *Enteromorpha prolifera* (green alga), Jinjiang Saltern, Fujian province, China	[70]
6-*O*-α-D-Ribosyl-8-*O*-methylasperentin (**181**)	438	C_22_H_30_O_9_	F00785, *Enteromorpha prolifera* (green alga), Jinjiang Saltern, Fujian province, China	[70]
5′-Hydroxyasperentin (**182**)	308	C_16_H_20_O_6_	F00785, *Enteromorpha prolifera* (green alga), Jinjiang Saltern, Fujian province, China	[70]
4′-Hydroxyasperentin (**183**)	308	C_16_H_20_O_6_	F00785, *Enteromorpha prolifera* (green alga), Jinjiang Saltern, Fujian province, China	[70]
Asperentin-8-methyl ether (**184**)	306	C_17_H_22_O_5_	F00785, *Enteromorpha prolifera* (green alga), Jinjiang Saltern, Fujian province, China	[70]
5′-Hydroxyasperentin-8-methyl ether (**185**)	322	C_17_H_22_O_6_	F00785, *Enteromorpha prolifera* (green alga), Jinjiang Saltern, Fujian province, China	[70]
4′-Hydroxyasperentin-6-methyl ether (**186**)	322	C_17_H_22_O_6_	F00785, *Enteromorpha prolifera* (green alga), Jinjiang Saltern, Fujian province, China	[70]
(3*R*,4*S*)-3,4,5-Trimethyl-isochroman-6,8-diol (**187**)	208	C_12_H_16_O_3_	YH11-2, deep-sea fungus, Guam, South Japan	[44]
(3*R*,4*S*)-6,8-dihydroxy-3,4,5-trimethylisochroman-1-one (**188**)	222	C_12_H_14_O_4_	YH11-2, deep-sea fungus, Guam, South Japan	[44]
2-(12*S*-Hydroxypropyl)-3-hydroxymethyl-6-hydroxy-7-methoxychromone (**189**)	280	C_14_H_16_O_6_	#2B, *Aricennia marina* (leaves), Yangjiang, Guangdong, China	[64]
7-Hydroxy-2-(2-hydroxypropyl)-5-methyl chromone (**190**)	234	C_13_H_14_O_4_	J05B-7F-4, *Stelletta* sp. (marine sponge), South Korea	[36]
Aspercoumarine acid (**191**)	206	C_10_H_6_O_5_	MCCC 3A00324, deep-sea sediment, South Atlantic Ocean	[60]
(2*R*)-2,3-Dihydro-7-hydroxy-6, 8-dimethyl-2-[(E)-prop-1-enyl]chromen-4-one (**192**)	232	C_14_H_16_O_3_	YH11-2, deep-sea fungus, Guam, South Japan	[44]

**Table 7:** Chromane and coumarin derivatives reported from *Aspergillus sydowii* (molecular weight and formulae, strain, host, and location).

Compound Name	Mol. Wt.	Mol. Formula	Strain, Host, Location	Ref.
4-Hydroxy-3,6-dimethyl-2-pyrone (**194**)	140	C_7_H_8_O_3_	SCSIO 41301, *Phakellia fusca* (marine sponge), Xisha Islands, China	[35]
4-Methyl-5,6-dihydropyren-2-one (**193**)	112	C_6_H_8_O_2_	SCSIO 41301, *Phakellia fusca* (marine sponge), Xisha Islands, China	[35]
Sydowione A (**195**)	226	C_12_H_18_O_4_	SCSIO 00305, *Verrucella unbracculum* (gorgonian), South China Sea, Sanya, Hainan, China	[24]
Sydowione B (**196**)	226	C_12_H_18_O_4_	SCSIO 00305, *Verrucella unbracculum* (gorgonian), South China Sea, Sanya, Hainan, China	[24]
Paecilpyrone A (**197**)	238	C_13_H_18_O_4_	SCSIO 00305, *Verrucella unbracculum* (gorgonian), South China Sea, Sanya, Hainan, China	[24]
(±)-Pyrenocine S (**198**)	226	C_11_H_14_O_5_	#2B, *Aricennia marina* (leaves), Yangjiang, Guangdong, China	[64]
Pyrenocine A (**199**)	208	C_11_H_12_O_4_	#2B, *Aricennia marina* (leaves), Yangjiang, Guangdong, China	[64]
(±)-Pyrenocine E (**200**)	240	C_12_H_16_O_5_	#2B, *Aricennia marina* (leaves), Yangjiang, Guangdong, China	[64]
Asperphenylpyrone (**201**)	310	C_18_H_14_O_5_	MCCC 3A00324, deep-sea sediment, South Atlantic Ocean	[60]
Macrolactin U′ (**202**)	480	C_31_H_44_O_4_	Deep-sea mud, Dalian, China	[58]
Sydocyclopropane A (**203**)	270	C_14_H_22_O_5_	MCCC 3A00324, deep-sea sediment, South Atlantic Ocean	[42]
Sydocyclopropane B (**204**)	182	C_11_H_18_O_2_	MCCC 3A00324, deep-sea sediment, South Atlantic Ocean	[42]
Sydocyclopropane C (**205**)	184	C_10_H_16_O_3_	MCCC 3A00324, deep-sea sediment, South Atlantic Ocean	[42]
Sydocyclopropane D (**206**)	184	C_10_H_16_O_3_	MCCC 3A00324, deep-sea sediment, South Atlantic Ocean	[42]
Hamavellone B (**207**)	180	C_11_H_16_O_2_	MCCC 3A00324, deep-sea sediment, South Atlantic Ocean	[42]
Sydowione C (**208**)	284	C_15_H_24_O_5_	SCSIO 00305, *Verrucella unbracculum* (gorgonian), South China Sea, Sanya, Hainan, China	[24]
Cycloerodiol (**209**)	240	C_15_H_28_O_2_	Cultured, China	[28]
Sydowin A (**210**)	412	C_18_H_14_Cl_2_O_7_	*Acanthophora spicifera* (red alga), Rameswaram, India	[53]
Sydowin B (**211**)	396	C_18_H_14_Cl_2_O_6_	*Acanthophora spicifera* (red alga), Rameswaram, India	[53]
3-(2-Hydroxypropyl)-4-(hexa-2*E*,4*E*-dien-6-yl)furan-2(5*H*)-one (**212**)	222	C_13_H_18_O_3_	Cultured, China	[28]
Pestalotiolactone A (**213**)	184	C_10_H_16_O_3_	MCCC 3A00324, deep-sea sediment, South Atlantic Ocean	[60]
1-Hydroxyboivinianin A (**214**)	206	C_12_H_14_O_3_	MCCC 3A00324, deep-sea sediment, South Atlantic Ocean	[57]
(±)-Sydowiccal (**215**)	222	C_12_H_14_O_4_	*Rhododendron mole* (leaves), Xing’an, Guangxi, China	[26]
Butyrolactone-I (**216**)	424	C_24_H_24_O_7_	#2B, *Aricennia marina* (leaves), Yangjiang, Guangdong, China	[64]

**Table 8:** Other metabolites reported from *Aspergillus sydowii* (molecular weight and formulae, strain, host, and location).

Compound Name	Mol. Wt.	Mol. Formula	Strain, Host, Location	Ref.
Sydowether (**217**)	354	C_18_H_26_O_7_	SW9, seawater, Yangma Island, Yantai, China	[41]
1,9-Dihydroxy-3-(hydroxymethyl)-10-methoxydibenzo[b,e]oxepine- 6,11-dione (**218**)	316	C_16_H_12_O_7_	*Scapania ciliata* (Chinese liverwort), Maoer Mountain, Guangxi, China	[63]
8-Demethoxy-10-methoxy-wentiquinone C (**219**)	300	C_16_H_12_O_6_	C1-S01-A7, seawater, West Pacific Ocean	[55]
Moniliphenone (**220**)	286	C_16_H_14_O_5_	*Scapania ciliata* (Chinese liverwort), Maoer Mountain, Guangxi, China	[63]
Phenol A acid (**221**)	240	C_12_H_16_O_5_	EN-534, *Laurencia okamurai* (red alga), Qingdao, China	[69]
Phenol A (**222**)	196	C_11_H_16_O_3_	EN-534, *Laurencia okamurai* (red alga), Qingdao, China	[69]
3-(2,5-Dimethylbenzo[d][1,3]dioxol-2-yl)propanoic acid (**223**)	222	C_12_H_14_O_4_	SCSIO 41301, *Phakellia fusca* (marine sponge), Xisha Islands, China	[35]
2-(5-Hydroxy-4-methylpentyl)-2-methylbenzo[d][1,3]dioxole-5- carboxylic acid (**224**)	280	C_15_H_20_O_5_	SCSIO 41301, *Phakellia fusca* (marine sponge), Xisha Islands, China	[35]
4-Hydroxyphenylacetic acid (**225**)	152	C_8_H_8_O_3_	SP-1, marine sediment, Antarctic Great Wall Station	[40]
Orcinol (**226**)	124	C_7_H_8_O_2_	PSU-F154, genus *Annella* sp. (gorgonian sea fan), coastal area, Surat Thani, Thailand	[56]
3-Hydroxybenzoic acid (**227**)	138	C_7_H_6_O_3_	CPCC 401353, cultured, China	[59]
4-Hydroxybenzoic acid (**228**)	138	C_7_H_6_O_3_	MCCC 3A00324, deep-sea sediment, South Atlantic Ocean	[60]
3,4,5-Trimethoxybenzoic acid (**229**)	212	C_10_H_12_O_5_	MCCC 3A00324, deep-sea sediment, South Atlantic Ocean	[60]
4-(3′,4′-Dihydroxyphenyl)-2-butanone (**230**)	180	C_10_H_12_O_3_	MCCC 3A00324, deep-sea sediment, South Atlantic Ocean	[60]
4-Hydroxybenzaldehyde (**231**)	122	C_7_H_6_O_2_	C1-S01-A7, seawater, West Pacific Ocean	[55]
Benzoic acid (**232**)	122	C_7_H_6_O_2_	CPCC 401353, cultured, China	[59]
Gibellulin B (**233**)	260	C_14_H_12_O_5_	FNA026, seawater, Xiamen, China	[9]
3,7-Dihydroxy-1,9-dimethyldibenzofuran (**234**)	228	C_14_H_12_O_3_	FNA026, seawater, Xiamen, China	[9]
	-	-	SCSIO 41301, *Phakellia fusca* (marine sponge), Xisha Islands, China	[35]
	-	-	MCCC 3A00324, deep-sea sediment, South Atlantic Ocean	[60]
Orsellinic acid (**235**)	168	C_8_H_8_O_4_	Deep-sea mud, Dalian, China	[58]
	-	-	CPCC 401353, cultured, China	[59]
2-Methoxy-5-methyl-3-(methylsulfonyl)phenol (**236**)	216	C_9_H_12_O_4_S	*Rhododendron mole* (leaves), Xing’an, Guangxi, China	[26]
2,3,5-Trimethyl-6-(3-oxobutan-2-yl)-4H-pyran-4-one (**237**)	208	C_12_H_16_O_3_	YH11-2, deep-sea fungus, Guam, South Japan	[44]
5-[(2*S*,3*R*)-3-Hydroxybutan-2-yl]-4-methylbenzene-1,3-diol (**238**)	196	C_11_H_16_O_3_	YH11-2, deep-sea fungus, Guam, South Japan	[44]
2,4-Dihydroxy-3,5,6-trimethylbenzaldehyde (**239**)	180	C_10_H_12_O_3_	YH11-2, deep-sea fungus, Guam, South Japan	[44]
Macrolactin A (**240**)	402	C_24_H_34_O_5_	Piece of deep-sea mud, Dalian, China	[58]
(*Z*)-6-Tridecenoic acid (**241**)	212	C_13_H_24_O_2_	Cultured, China	[28]
Malvidin 3-O-glucoside (**242**)	479	C_22_H_23_O_12_^+\^	H-1, bacterial wilt-affected ginger humus, Chengdu, China	[25]
Malvidin 3-O-galactoside (**243**)	449	C_21_H_21_O_11_^+^	H-1, bacterial wilt-affected ginger humus, Chengdu, China	[25]
Cyanidin 3-O-glucoside (**244**)	493	C_23_H_25_O_12_^+^	H-1, bacterial wilt-affected ginger humus, Chengdu, China	[25]
Peonidin O-malonylhexoside (**245**)	549	C_25_H_25_O_14_^+^	H-1, bacterial wilt-affected ginger humus, Chengdu, China	[25]
Cyanidin (**246**)	287	C_15_H_11_O_6_^+^	H-1, bacterial wilt-affected ginger humus, Chengdu, China	[25]

**Table 9:** Cytotoxic metabolites reported from *A. sydowii.*

Compound Name	Assay/Cell Line	Biological Results (IC_50_) *		Ref.
		Compound	Positive Control	
Cerevisterol (**67**)	P388/SRB	0.12 μM	CDDP 0.039 μM	[44]
6-Methoxyl austocystin A (**83**)	*Artemia salina*	2.9 µM	Toosendanin 2.2 µM	[24]
[4-(2-Methoxyphenyl)-1-piperazinyl][(1-methyl-1*H*-indol-3-yl)]-methanone (**117**)	MTT/A375	5.7 μM	-	[31]
(3*R*,4*S*)-3,4,5-Trimethylisochroman-6,8-diol (**187**)	P388/SRB	1.95 μM	CDDP 0.039 μM	[44]
(2*R*)-2,3-Dihydro-7-hydroxy-6,8-dimethyl-2-[(*E*)-prop-1-enyl] chromen-4-one (**192**)	P388/SRB	0.14 μM	CDDP 0.039 μM	[44]
Sydowione A (**195**)	*Artemia salina*	19.5 µM	Toosendanin 2.2 µM	[24]
Sydowione B (**196**)	*Artemia salina*	14.3 µM	Toosendanin 2.2 µM	[24]
Sydowione C (**208**)	*Artemia salina*	8.3 µM	Toosendanin 2.2 µM	[24]
2,4-Dihydroxy-3,5,6-trimethylbenzaldehyde (**239**)	P388/SRB	0.59 μM	CDDP 0.039 μM	[44]

* IC50, Half maximal inhibitory concentration.

**Table 10:** Anti-mycobacterial, antimicrobial, and anti-microalgal metabolites reported from *A. sydowii.*

Compound Name	Assay/Organism	Biological Results		Ref.
		Compound	Positive Control	
**Antibacterial (MIC)**				
(7*S*)-(+)-hydroxysydonic acid (**2**)	Broth microdilution/*S. aureus*	0.5 µg/mL	Tigecycline 0.06 µg/mL	[40]
	Broth microdilution/ MRSA	1 µg/mL	Tigecycline 0.25 µg/mL	[40]
	Broth microdilution/*S. epidermidis*	0.25 µg/mL	Tigecycline 0.03 µg/mL	[40]
	Broth microdilution/ MRAE	0.5 µg/mL	Tigecycline 0.12 µg/mL	[40]
(7*S*,11*S*)-(+)-12-Hydroxysydonic acid (**5**)	Broth microdilution/*S. aureus*	0.5 µg/mL	Tigecycline 0.06 µg/mL	[40]
	Broth microdilution/ MRSA	1 µg/mL	Tigecycline 0.25 µg/mL	[40]
	Broth microdilution/*S. epidermidis*	0.25 µg/mL	Tigecycline 0.03 µg/mL	[40]
	Broth microdilution/ MRAE	0.5 µg/mL	Tigecycline 0.12 µg/mL	[40]
(11*S*,14*S*)-Cyclo-(L-Trp-L-Phe) (**110**)	Broth microdilution/*S. aureus*	0.25 µg/mL	Tigecycline 0.06 µg/mL	[40]
	Broth microdilution/ MRSA	1 µg/mL	Tigecycline 0.25 µg/mL	[40]
	Broth microdilution/*S. epidermidis*	0.12 µg/mL	Tigecycline 0.03 µg/mL	[40]
	Broth microdilution/ MRAE	0.5 µg/mL	Tigecycline 0.12 µg/mL	[40]
Citrinin (**169**)	Microplate assay/*E. coli*	8 µg/mL	Chloramphenicol 1 µg/mL	[69]
	Microplate assay/*Micrococcus luteus*	16 µg/mL	Chloramphenicol 2 µg/mL	[69]
	Microplate assay/*Vibrio parahaemolyticus*	8 µg/mL	Chloramphenicol 2 µg/mL	[69]
Penicitrinol A (**170**)	Microplate assay/*E. coli*	8 µg/mL	Chloramphenicol 1 µg/mL	[69]
	Microplate assay/*Micrococcus luteus*	4 µg/mL	Chloramphenicol 2 µg/mL	[69]
	Microplate assay/*Vibrio parahaemolyticus*	8 µg/mL	Chloramphenicol 2 µg/mL	[69]
Antituberculosis (IC_50_)				
Sydowiol A (**166**)	*M. tuberculosis* protein tyrosine phosphatase inhibitor	14.0 μg/mL	-	[37]
Sydowiol B (**167**)		24.0 μg/mL	-	[37]
Anti-microalgae (IC_50_)				
(7*S*)-Flavilane A (**53**)	Broth microdilution/ *Prorocentrum micans*	4.6 µg/mL	CuSO_4_ 2.7 µg/mL	[38]
	Broth microdilution/ *Prorocentrum minimum*	2.4 µg/mL	CuSO_4_ 2.2 µg/mL	[38]
(7*S*)- 4-Iodo-flavilane A (**54**)	Broth microdilution/ *Prorocentrum micans*	11.0 µg/mL	CuSO_4_ 2.7 µg/mL	[38]
	Broth microdilution/ *Prorocentrum minimum*	1.3 µg/mL	CuSO_4_ 2.2 µg/mL	[38]
Bisviolaceol II (**165**)	Broth microdilution/ *Prorocentrum minimum*	5.2 µg/mL	CuSO_4_ 2.2 µg/mL	[38]

**Table 11:** Anti-influenza virus metabolites reported from *Aspergillus sydowii.*

Compound Name	Virus/Assay	Biological Results (IC_50_)		Ref.
		Compound	Positive Control	
7-Deoxy-7,14-didehydrosydonic acid (**8**)	Puerto Rico/8/34 (H1N1)/Pseudovirus neutralization and MTT	7.07 µM	Ribavirin 2.53 µM	[35]
cyclo-12-Hydroxysydonic acid (**22**)	Puerto Rico/8/34 (H1N1)/Pseudovirus neutralization and MTT	8.89 µM	Ribavirin 2.53 µM	[35]
	Aichi/2/68 (H3N2)/Pseudovirus neutralization and MTT	36.41 µM	Ribavirin 6.23 µM	[35]
	FM-1/1/47(H1N1)/Pseudovirus neutralization and MTT	24.46 µM	Ribavirin 3.97 µM	[35]
2-Hydroxy-1-(hydroxymethyl)-8-methoxy-3-methyl-9*H*-xanthen-9-one (**80**)	Puerto Rico/8/34 (H1N1)/Pseudovirus neutralization and MTT	4.70 µM	Ribavirin 2.53 µM	[35]
	FM-1/1/47 (H1N1)/Pseudovirus neutralization and MTT	4.04 µM	Ribavirin 3.97 µM	[35]
2-Hydroxy-1-(hydroxymethyl)-7,8-dimethoxy-3-methyl-9*H*-xanthen-9-one (**81**)	Puerto Rico/8/34 (H1N1)/Pseudovirus neutralization and MTT	2.17 µM	Ribavirin 2.53 µM	[35]
Emodic acid (**92**)	Puerto Rico/8/34 (H1N1)/Pseudovirus neutralization and MTT	2.00 µM	Ribavirin 2.53 µM	[35]
	Aichi/2/68 (H3N2)/Pseudovirus neutralization and MTT	17.53 µM	Ribavirin 6.23 µM	[35]
	FM-1/1/47(H1N1)/Pseudovirus neutralization and MTT	5.37 µM	Ribavirin 3.97 µM	[35]
Parietinic acid (**93**)	Puerto Rico/8/34 (H1N1)/Pseudovirus neutralization and MTT	7.88 µM	Ribavirin 2.53 µM	[35]
	Aichi/2/68 (H3N2)/Pseudovirus neutralization and MTT	30.09 µM	Ribavirin 6.23 µM	[35]
	FM-1/1/47(H1N1)/Pseudovirus neutralization and MTT	39.60 µM	Ribavirin 3.97 µM	[35]
Questin (**94**)	Puerto Rico/8/34 (H1N1)/Pseudovirus neutralization and MTT	1.92 µM	Ribavirin 2.53 µM	[35]
	Aichi/2/68 (H3N2)/Pseudovirus neutralization and MTT	9.62 µM	Ribavirin 6.23 µM	[35]
	FM-1/1/47(H1N1)/Pseudovirus neutralization and MTT	11.1 µM	Ribavirin 3.97 µM	[35]
1,6,8-Trihydroxy-3-methylanthraquinone (**95**)	Aichi/2/68 (H3N2)/Pseudovirus neutralization and MTT	9.72 µM	Ribavirin 6.23 µM	[35]
	FM-1/1/47(H1N1)/Pseudovirus neutralization and MTT	18.48 µM	Ribavirin 3.97 µM	[35]
Bisdethiobis(methylthio)-acetylaranotin (**116**)	Puerto Rico/8/34 (H1N1)/Pseudovirus neutralization and MTT	34.60 µM	Ribavirin 2.53 µM	[35]
	Aichi/2/68 (H3N2)/Pseudovirus neutralization and MTT	24.56 µM	Ribavirin 6.23 µM	[35]
	FM-1/1/47(H1N1)/Pseudovirus neutralization and MTT	44.08 µM	Ribavirin 3.97 µM	[35]
Citrinin (**169**)	H_5_N_1_/Influenza neuraminidase inhibition screen kit	45.6 nM	Oseltamivir 3.6 nM	[69]
Penicitrinol A (**170**)	H_5_N_1_/Influenza neuraminidase inhibition screen kit	21.2 nM	Oseltamivir 3.6 nM	[69]
seco-Penicitrinol A (**171**)	H_5_N_1_/Influenza neuraminidase inhibition screen kit	24.7 nM	Oseltamivir 3.6 nM	[69]
Penicitrinol L (**172**)	H_5_N_1_/Influenza neuraminidase inhibition screen kit	41.5 nM	Oseltamivir 3.6 nM	[69]
Penicitrinone A (**173**)	H_5_N_1_/Influenza neuraminidase inhibition screen kit	12.9 nM	Oseltamivir 3.6 nM	[69]
Penicitrinone F (**174**)	H_5_N_1_/Influenza neuraminidase inhibition screen kit	18.5 nM	Oseltamivir 3.6 nM	[69]
Sydocyclopropane A (**203**)	WSN/33 (H1N1)/Cytopathic effect reduction/A/WSN/33 (H1N1)	26.7 μM	Oseltamivir 18.1 μM	[42]
Sydocyclopropane B (**204**)	Cytopathic effect reduction/A/WSN/33 (H1N1)	29.5 μM	Oseltamivir 18.1 μM	[42]
Hamavellone B (**207**)	Cytopathic effect reduction/A/WSN/33 (H1N1)	35.8 μM	Oseltamivir 18.1 μM	[42]
3,7-Dihydroxy-1,9-dimethyldibenzofuran (**234**)	Puerto Rico/8/34 (H1N1)/Pseudovirus neutralization and MTT	1.31 µM	Ribavirin 2.53 µM	[35]
	Aichi/2/68 (H3N2)/Pseudovirus neutralization and MTT	1.24 µM	Ribavirin 6.23 µM	[35]
	FM-1/1/47(H1N1)/Pseudovirus neutralization and MTT	2.84 µM	Ribavirin 3.97 µM	[35]

**Table 12:** Anti-inflammatory metabolites reported from *Aspergillus sydowii.*

Compound Name	Assay	Biological Results		Ref.
		Compound	Positive Control	
(S)-(+)-Sydonic acid (**1**)	Inhibition of superoxide anion	17.82 μM (IC_50_)	Sorafenib 1.27 μM (IC_50_)	[54]
(7*S*,11*S*)-(+)-12-Hydroxysydonic acid (**5**)	Inhibition of superoxide anion	31.95 μM (IC_50_)	Sorafenib 1.27 μM (IC_50_)	[54]
Aspergillusene A (**42**)	Inhibition of superoxide anion	6.11 μM (IC_50_)	Sorafenib 1.27 μM (IC_50_)	[54]
	Inhibition of elastase release	8.80 μM (IC_50_)	Sorafenib 1.27 μM (IC_50_)	[54]
(+)-(7*S*)-Sydonol (**45**)	Inhibition of superoxide anion	5.23 μM (IC_50_)	Sorafenib 1.27 μM (IC_50_)	[54]
	Inhibition of elastase release	16.39 μM (IC_50_)	Sorafenib 1.27 μM (IC_50_)	[54]
(7*S*)-(+)-7-O-Methylsydonol (**46**)	Inhibition of superoxide anion	13.80 μM (IC_50_)	Sorafenib 1.27 μM (IC_50_)	[54]
Anhydrowaraterpol B (**49**)	Inhibition of superoxide anion	21.52 μM (IC_50_)	Sorafenib 1.27 μM (IC_50_)	[54]
Sydowinin B (**71**)	Inhibition of superoxide anion	21.20 μM (IC_50_)	Sorafenib 1.27 μM (IC_50_)	[54]
	Inhibition of elastase release	12.62 μM (IC_50_)	Sorafenib 1.27 μM (IC_50_)	[54]
(7*R*,8*R*)-AGI-B4 (**88**)	Inhibition of superoxide anion	6.00 μM (IC_50_)	Sorafenib 1.27 μM (IC_50_)	[54]
	Inhibition of elastase release	6.60 μM (IC_50_)	Sorafenib 1.27 μM (IC_50_)	[54]

## Data Availability

Not applicable.
